# Machine learning-driven ultrasound radiomics for assessing axillary lymph node burden in breast cancer

**DOI:** 10.3389/fendo.2025.1548888

**Published:** 2025-02-27

**Authors:** Si-Rui Wang, Feng Tian, Tong Zhu, Chun-Li Cao, Jin-Li Wang, Wen-Xiao Li, Jun Li, Ji-Xue Hou

**Affiliations:** ^1^ The Ultrasound Diagnosis Department of the First Affiliated Hospital of Shihezi University, Shihezi, Xinjiang, China; ^2^ The Neurology Department of the First Affiliated Hospital of Shihezi University, Shihezi, Xinjiang, China; ^3^ The Thyroid and Breast Surgery Department of the First Affiliated Hospital of Shihezi University, Shihezi, Xinjiang, China

**Keywords:** breast cancer, ultrasound, axillary lymph nodes burden, radiomics, machine learning

## Abstract

**Objective:**

This study explores the value of combining intratumoral and peritumoral radiomics features from ultrasound imaging with clinical characteristics to assess axillary lymph node burden in breast cancer patients.

**Methods:**

A total of 131 breast cancer patients with axillary lymph node metastasis (ALNM) were enrolled between June 2019 and September 2024. Patients were divided into low (n=79) and high (n=52) axillary lymph node burden (ALNB) groups. They were further split into training (n=92) and validation (n=39) cohorts. Intratumoral and peritumoral features were analyzed using the maximum relevance minimum redundancy (MRMR) and least absolute shrinkage and selection operator (LASSO) methods. Six machine learning models were evaluated, and a combined clinical-radiomics model was built.

**Results:**

The combined logistic regression model exhibited superior diagnostic performance for high axillary lymph node burden, with areas under the ROC curve (AUC) of 0.857 in the training cohort and 0.820 in the validation cohort, outperforming individual models. The model balanced sensitivity and specificity well at a 52% cutoff value. A nomogram provided a practical risk assessment tool for clinicians.

**Conclusion:**

The combined clinical-radiomics model showed excellent predictive ability and may aid in optimizing management and treatment decisions for breast cancer patients.

## 1 Introduction

The status and extent of axillary lymph node (ALN) metastasis are critical prognostic factors for the recurrence and survival of breast cancer patients (1). Early and accurate assessment of lymph node involvement is essential for axillary staging and the development of an appropriate treatment strategy. Currently, it is widely accepted that tumor cells typically first spread from the primary site to nearby regional lymph nodes and then metastasize to distant organs. Thus, axillary lymph node dissection (ALND) (2) is considered the standard treatment for early breast cancer patients with positive ALNs. However, as an invasive procedure, ALND is associated with complications such as lymphedema, infection, restricted shoulder movement, and neurovascular injury, all of which negatively impact the quality of life for patients.

With advances in breast cancer treatment, sentinel lymph node biopsy (SLNB) has emerged as a safer, less invasive, and diagnostically accurate alternative (3). By selectively detecting the first group of lymph nodes that drain from the primary tumor, SLNB effectively monitors whether axillary lymph node metastasis (ALNM) has occurred, thereby avoiding the need for extensive dissection. SLNB not only provides an accurate assessment of ALN status but also significantly reduces surgery-related complications. Today, SLNB has become the standard method for axillary lymph node staging in breast cancer patients (4). However, there remains clinical controversy over whether patients with positive sentinel lymph nodes require axillary clearance. Some studies have shown that in patients with preoperative lymph node biopsies showing positivity, only 1-2 metastatic lymph nodes were found in the ALND specimens (5, 6). According to guidelines from the American Society for Clinical Oncology (ASCO) (7), early-stage breast cancer patients with T1-T2 disease and 1-2 positive sentinel lymph nodes can forgo ALND in favor of breast-conserving surgery combined with whole-breast irradiation. The long-term follow-up data from the Z0011 trial revealed that the 5-year incidence of postoperative lymphedema in the SLNB group was only 6%, significantly lower than the 25% observed in the ALND group. Additionally, patients in the SLNB group experienced faster recovery of shoulder joint function and lower postoperative pain scores (8, 9). This demonstrates that for patients with low axillary lymph node metastatic burden, SLNB not only avoids unnecessary trauma but also significantly improves postoperative quality of life. Therefore, for T1-T2 breast cancer patients with low axillary lymph node metastasis burden, routine ALND may not be necessary for postoperative treatment. Despite its high diagnostic accuracy, Studies indicate that the false-negative rate of SLNB is approximately 5–10%, particularly in cases involving small metastatic foci (<2 mm) or skip metastases. Such missed diagnoses may lead to inaccurate staging in patients with high lymph node burden, thereby affecting adjuvant treatment decisions. To address this challenge, clinical guidelines currently recommend the use of dual tracers (e.g., blue dye combined with radioactive colloid) to improve SLN detection rates but use of dual tracers for lymph node tracing may cause allergic reactions in some patients. Consequently, there is a pressing need to develop a safe and effective non-invasive technique that can accurately assess the ALNM burden preoperatively, offering a potential alternative to SLNB and reducing postoperative complications from ALND.

Ultrasound, with its advantages of being simple, fast, and non-invasive, has been widely used in the detection of breast tumors. The introduction of the Breast Imaging-Reporting and Data System (BI-RADS) has further standardized the description of breast masses for malignancy. However, ultrasound has limitations in detecting ALNM (10). Studies have shown that when the number of metastatic lymph nodes is fewer than three, ultrasound sensitivity and specificity significantly decrease, leading to potential missed or inaccurate diagnoses. Moreover, diagnostic accuracy among ultrasound physicians can vary considerably due to differences in experience and scanning habits. Results from Cui et al. (11) indicated that the diagnostic efficiency of less-experienced ultrasound physicians was 20% lower compared to that of more experienced physicians.

Radiomics, a rapidly evolving imaging analysis technique, has been widely applied in disease diagnosis, prognosis, and treatment efficacy prediction. This technique extracts a large number of quantitative imaging features, selects and reduces dimensionality, and reveals characteristics that make non-invasive precision medicine possible. Using automated data characterization algorithms, radiomics can extract and select optimal features from regions of interest (ROIs) that often have high diagnostic value in breast cancer masses, including features based on grayscale, texture, and shape, which are then quantified. These features reflect biological information about the tumor and are highly correlated with disease status (12, 13). Recent studies have demonstrated (14, 15) that machine learning models based on ultrasound radiomics exhibit significant advantages in predicting high axillary lymph node burden(AHNB) (defined as more than two metastatic lymph nodes), with sensitivity and specificity substantially higher than those of conventional ultrasound. By extracting features such as gray-level co-occurrence matrix (GLCM) and wavelet transform characteristics from tumor regions, these models can reveal spatial heterogeneity in the tumor microenvironment, capturing potential biological behaviors associated with lymph node metastasis. Although further external validation is required to confirm their generalizability, the non-invasive nature and real-time analysis potential of these models position them as promising auxiliary tools for preoperative assessment.

This study preliminarily screened clinical-pathological features and radiomics features associated with high lymph node burden through univariate analysis. Independent risk factors were subsequently identified using LASSO regression and multivariate logistic regression. Model performance was evaluated via 5-fold cross-validation and independent external cohort validation, with discrimination ability assessed using AUC, sensitivity, and specificity. Clinical utility was further validated through calibration curves and decision curve analysis (DCA). The final integrated model combines key independent risk factors to enable personalized prediction.

## 2 Methods

### 2.1 Study population

This study prospectively and retrospectively collected 131 cases of T1-T2 stage primary unifocal breast cancer with axillary lymph node metastasis (ALNM) from June 2019 to September 2024 at the First Affiliated Hospital of Shihezi University. Among them, 79 patients were confirmed with low axillary lymph node burden (ALNB) (≤2 metastatic lymph nodes), with an average age of 55.99 ± 11.05 years (**Table 1**), 52patients were confirmed with AHNB (>2 metastatic lymph nodes), with an average age of 54.10 ± 9.56 years. All patients had not received any treatment before surgical resection and had complete preoperative ultrasound images and clinicopathological data. The cases were randomly divided into a training group (n=92) and a validation group (n=39) in a 7:3 ratio. This study was approved by the Medical Ethics Committee of the hospital (KJ2023-412-01), and all patients provided written informed consent before surgery.

**Table 1:** Pathological types of patients with axillary lymph node metastases from breast cancer included.

Pathology	No. (%) of patients	
	ALNB	AHNB
Ductal Carcinoma in Situ	4 (5.1%)	7 (13.5%)
Intraductal Papillary Carcinoma	0 (0%)	1 (1.9%)
Invasive Ductal Carcinoma	72 (91.1%)	41 (78.8%)
Invasive Lobular Carcinoma	1 (1.3%)	0 (0%)
Mucinous Carcinoma	0 (0%)	0 (0%)
Medullary Carcinoma	0 (0%)	0 (0%)
Mixed Breast Carcinoma	2 (2.5%)	3 (5.8%)
Total	79	52

1) Inclusion Criteria:

a. Female patients aged 18-90 years.

b. All patients diagnosed with breast cancer via postoperative pathology or core needle biopsy, with clear lymph node pathology diagnosis.

c. Availability of clear and complete preoperative ultrasound images of the breast mass.

d. Patients consented to participate in the study.

2) Exclusion Criteria:

a. Patients who underwent core needle biopsy or interventional treatment before ultrasound examination.

b. Breast masses with unclear boundaries on ultrasound or with a diameter greater than 5 cm.

c. Patients with multifocal or multicentric breast cancer confirmed by pathology.

d. Incomplete clinical information (either clinical data or ultrasound images) or without a clear pathological diagnosis.

e. Patients with other tumors or a history of recurrent breast cancer.

### 2.2 Equipment and methods

#### 2.2.1 Ultrasound equipment

Ultrasound machines used in this study included Siemens S3000 (Germany), SAMSUNG RS85, SAMSUNG RS80, and SAMSUNG R10 (South Korea) color Doppler ultrasound diagnostic systems, equipped with 9L4 (4-9 MHz) and L3-12a (3-12 MHz) linear probes. All ultrasound examinations were performed by uniformly trained physicians using standardized device parameters (gain: 50–60 dB, depth: 4–6 cm, frequency: 8–12 MHz). Image quality was independently assessed by two senior physicians, excluding low-quality images with motion artifacts or uneven probe pressure (e.g., blurred boundaries or absent blood flow signals).

#### 2.2.2 Ultrasound examination

Patients were positioned supine with their hands above their heads to fully expose both breasts and axillary regions. They were instructed to breathe calmly. Scanning was conducted in a counterclockwise direction, starting from the upper outer quadrant, lower outer quadrant, lower inner quadrant, and upper inner quadrant, covering the entire breast in an overlapping pattern.

#### 2.2.3 Ultrasound image feature extraction

The ultrasound images were independently assessed by two ultrasound physicians, each with over ten years of experience, using a double-blind method. The ultrasound image characteristics of breast masses in enrolled patients included maximum lesion diameter (≤2 or >2 cm), tumor location (upper inner quadrant, lower inner quadrant, upper outer quadrant, lower outer quadrant, or periareolar region), aspect ratio (≤1 or >1), internal echo (homogeneous or heterogeneous), posterior acoustic attenuation (absent or present), margin clarity (clear or unclear), margin angulation (absent or present), morphology (regular or irregular), microcalcifications (absent or present), Adler blood flow grading (Grade 0, I, II, or III), and blood flow resistance index (RI). In cases of disagreement between the two physicians, the final result was determined through consultation or referral to a senior physician.

### 2.3 Image segmentation and feature selection

An ultrasound physician with over 10 years of experience in diagnosing breast diseases used 3D-Slicer software to outline the region of interest (ROI) of the tumor (intra-tumoral) on conventional ultrasound images (**Appendix Figures 1-1A, 1-1B**). Using morphological dilation techniques, the tumor perimeter was radially expanded by 1 to 5 mm according to pixel size to obtain the expanded peri-tumoral ROI (**Appendix Figures 1-2A, 1-2B**). Features from the conventional ultrasound intra-tumoral and peri-tumoral ROIs were extracted and filtered. Feature selection involved two steps: (1) MRMR (Maximum Relevance Minimum Redundancy) for initial screening of high-correlation, low-redundancy features, followed by (2) LASSO regression with 10-fold cross-validation for further dimensionality reduction. The area under the ROC curve (AUC) of six models from the intra-tumoral and peri-tumoral (1 to 5 mm) regions was compared to identify the optimal intra-tumoral + peri-tumoral model, and the DeLong test was used to compare AUC differences among the six models. The optimal model was selected based on sensitivity, specificity, and clinical net benefit (decision curve analysis). The final model required a significantly higher AUC (p<0.05) than other models, which was used to construct the Rad-score for the model.

### 2.4 Prediction model construction

Based on the pathology results, correlation analyses, univariate analysis, and LASSO regression were conducted to identify independent risk factors of AHNB from conventional ultrasound features and immunohistochemical markers. A clinical feature model for AHNB was then constructed. Subsequently, LASSO regression with 10-fold cross-validation was applied to further refine feature selection and identify independent risk factors from conventional ultrasound characteristics and immune-histochemical markers. These selected features were then integrated to construct a clinical feature model. To enhance predictive performance, the Rad-Score derived from the optimal intra-tumoral + peri-tumoral radiomics model was combined with the clinical features. Six machine learning algorithms—support vector machine (SVM), extreme gradient boosting (XGBoost), random forest (RF), logistic regression (LR), k-nearest neighbors (KNN), and decision tree (DT)—were trained and optimized using a nested 10-fold cross-validation framework. Hyperparameter tuning was implemented via grid search, with key parameters including SVM’s regularization term C (0.1, 1, 10), XGBoost’s maximum tree depth (3, 5, 7), and RF’s number of estimators (50, 100, 200). The diagnostic performance of the six algorithms was evaluated based on Average-AUC, Average-Accuracy, and Average-Kappa metrics from the 10-fold cross-validation, and the best machine learning method was selected using the area under the ROC curve. Additionally, calibration curves were used to assess model consistency, and the model was visualized based on the optimal algorithm to improve interpretability.

### 2.5 Feature consistency evaluation

In this study, conventional ultrasound images from 30 randomly selected breast cancer patients were used. Two physicians independently outlined the ROIs, and the intraclass correlation coefficient (ICC) was calculated to assess the reproducibility of radiomics feature extraction. The results showed good consistency (ICC > 0.8). For discordant cases (delineation difference >10%), a third senior physician arbitrated to determine the final ROI.

### 2.6 Clinical features

The positive reaction of estrogen receptors (ER) and progesterone receptors (PR) in the cell nucleus was indicated by brownish-yellow granules. A sample was considered positive if the proportion of positive cells exceeded 21%; otherwise, it was negative. For human epidermal growth factor receptor 2 (HER-2), positive cells on the cell membrane were indicated by brown granules. HER-2 was graded as 0, 1+, 2+, or 3+, with 3+ considered positive and 0 or 1+ negative. Samples graded as 2+ required further analysis using fluorescence *in situ* hybridization (FISH) to determine HER-2 gene amplification and confirm positive or negative status. Additionally, the Ki-67 proliferation index was considered low if the proportion of positive cells was less than 14% and high if it was 14% or greater.

The widely used Nottingham histological grading system for breast cancer was employed, which includes three components: ① the proportion of gland formation, ② nuclear pleomorphism, and ③ mitotic count. Each component was scored from 1 to 3, with the total score ranging from 3 to 9. A score of 3-5 indicated Grade 1 (G1), 6-7 was Grade 2 (G2), and 8-9 was Grade 3 (G3). Lymphovascular invasion was defined as the presence of breast cancer cells within lymphatic vessels or blood vessels stained positive for D2-40 and CD34 in the peri-tumoral area.

### 2.7 Statistical analysis

Data were analyzed using SPSS 22.0, R Studio 4.2.2, and Python (version 3.8) software, including clinical feature summary, radiomics feature selection, and generation of visualizations. Continuous variables were presented as mean ± standard deviation (x̅ ± s) or median (P25, P75), while categorical variables were expressed as frequencies (percentages). Differences between continuous variables were assessed using independent sample t-tests or the Wilcoxon rank-sum test. Differences between categorical variables were analyzed using the chi-square test or Fisher’s exact test, depending on the data distribution. The machine learning model analysis and construction were carried out using R language packages “e1071,” “xgboost,” “randomForest,” “stats,” “kknn,” and “rpart”.

## 3 Results

### 3.1 Clinical characteristics of patients

A total of 131 breast cancer patients with axillary lymph node metastasis (ALNM) were included in this study. This cohort comprised 11 cases of ductal carcinoma *in situ*, 1 case of intraductal papillary carcinoma, 113 cases of invasive ductal carcinoma, 1 case of invasive lobular carcinoma, and 5 cases of mixed-type breast cancer (**Table 1**). Among them, 79 patients (60.3%) were in the ALNB group, with an average age of 55.99 ± 11.05 years, while 52 patients (39.7%) were in the AHNB group, with an average age of 54.10 ± 9.56 years. Patients were randomly divided into a training group and a validation group at a 7:3 ratio. The training group included 92 patients (54 in the ALNB group (58.7%) and 38 in the AHNB group (41.3%)), while the validation group included 39 patients (25 in the ALNB group (48.7%) and 14 in the AHNB group (51.3%)).

**Table 2** presents the baseline ultrasound and clinicopathological characteristics of all enrolled patients. Univariate analysis revealed that Adler blood flow grading, RI, histological grade, and lymphovascular invasion were independent risk factors for AHNB (P < 0.05). Further correlation analysis demonstrated the relationships between these risk factors and other ultrasound and clinicopathological features (**Appendix Figure 2**). The correlation analysis highlighted moderate positive correlations between variables such as Adler blood flow grading, and these factors played significant roles in predicting AHNB. No statistically significant differences were observed between other ultrasound and clinicopathological features and AHNB (P > 0.05).

**Table 2:** Univariate analysis for predicting axillary lymph node load in breast cancer (overall group, training group and validation group).

Characteristics	Total (n=131)		*P* Value	*t/X^2^*	Training set (n=92)		*P* Value	*t/X^2^*	Validation set (n=39)		*P* Value	*t/X^2^*
	ALNB (n=79)	AHNB (n=52)			ALNB (n=54)	AHNB (n=38)			ALNB (n=25)	AHNB (n=14)		
**Age**	55.99±11.05	54.10±9.56	0.31	1.01	57.24±10.29	54.82±9.54	0.26	1.12	53.28±11.76	52.14±9.66	0.76	0.37
**Histological Grade**			0.52	0.42			0.42	0.66			0.79	0.07
G1/G2	38(48.1%)	22(42.3%)			23(42.6%)	13(34.2%)			15(60.0%)	9(64.3%)		
G3	41(51.9%)	30(57.7%)			31(57.4%)	25(65.8%)			10(40.0%)	5(35.7%)		
**Lymphovascular invasion**			<0.01	12.9			<0.01	9.07			0.04	3.92
No	30(38.0%)	5(9.6%)			21(38.9%)	4(10.5%)			9(36.0%)	1(7.1%)		
Yes	49(62.0%)	47(90.4%)			33(61.1%)	34(89.5%)			16(64.0%)	13(%)		
**ER**			0.89	0.60			0.67	1.56			0.45	2.63
0%-10%	13(16.5%)	10(19.2%)			9(16.7%)	9(23.7%)			4(16.0%)	1(7.1%)		
11%-40%	26(32.9%)	14(26.9%)			21(38.9%)	13(34.2%)			5(20.0%)	1(7.1%)		
41%-70%	21(26.6%)	14(26.9%)			11(20.4%)	5(13.2%)			10(40.0%)	9(64.3%)		
71%-100%	19(24.1%)	14(26.9%)			13(24.1%)	11(28.9%)			6(24.0%)	3(21.4%)		
**PR**			0.7	1.37			0.97	0.85			0.23	4.30
0%-10%	30(38.0%)	21(40.4%)			24(44.4%)	17(44.7%)			6(24.0%)	4(28.0%)		
11%-40%	12(15.2%)	5(9.6%)			8(14.8%)	5(13.2%)			4(16.0%)	0(0.0%)		
41%-70%	18(22.8%)	15(28.8%)			13(24.1%)	9(23.7%)			5(20.0%)	6(42.9%)		
71%-100%	19(24.1%)	11(21.2%)			9(16.7%)	7(18.4%)			10(40.0%)	4(28.6%)		
**HER-2**			0.68	0.12			0.77	0.10			0.58	0.30
-/+	35(44.3%)	23(44.2%)			23(42.6%)	15(39.5%)			12(48.0%)	8(57.1%)		
2+/3+	44(55.7%)	29(55.8%)			31(57.4%)	23(60.5%)			13(52.0%)	6(42.9%)		
**Ki-67**			0.69	0.16			0.31	1.02			0.43	0.61
≤14%	11(13.9%)	6(11.5%)			8(14.8%)	3(7.9%)			3(12.0%)	3(21.4%)		
>14%	68(86.1%)	46(88.5%)			46(85.2%)	35(92.1%)			22(88.0%)	11(78.6%)		
**Maximum lesion diameter**			0.01	6.38			0.04	4.42			0.04	4.73
≤2	42(533%)	16(30.8%)			29(53.7%)	12(31.6%)			13(52.0%)	4(28.6%)		
>2	37(46.8%)	36(69.2%)			25(46.3%)	26(68.4%)			12(48.0%)	10(71.4%)		
**Location**			0.56	2.99			0.40	4.06			0.57	2.91
Areola area	7(8.9%)	7(13.5%)			1(1.9%)	1(2.6%)			6(24.0%)	6(42.9%)		
Inner upper	14(17.7%)	9(17.3%)			11(20.4%)	9(23.7%)			3(12.0%)	0(0.0%)		
Inner lower	4(5.1%)	5(9.6%)			2(3.7%)	4(10.5%)			2(8.0%)	1(7.1%)		
Outer upper	40(50.6%)	26(50.0%)			29(53.7%)	21(55.3%)			11(44.0%)	5(35.7%)		
Outer lower	14(17.7%)	5(9.6%)			11(20.4%)	3(7.9%)			3(12.0%)	2(14.3%)		
**Orientation**			0.20	1.62			0.28	1.17			0.58	0.30
Horizontal	33(41.8%)	16(30.8%)			20(37.0%)	10(26.3%)			13(52.0%)	6(42.9%)		
Vertical	46(58.2%)	36(69.2%)			34(63.0%)	28(73.7%)			12(48.0%)	8(57.1%)		
**Echo pattern**			0.45	0.88			0.16	1.96			0.16	1.97
Homogeneous	5(6.3%)	8(15.4%)			1(1.9%)	3(7.9%)			4(16.0%)	5(35.7%)		
Inhomogeneous	74(93.7%)	44(84.6%)			53(98.1%)	35(92.1%)			21(84.0%)	9(64.3%)		
**Posterior acoustic decrease**			0.39	0.73			0.50	0.45			0.67	0.18
No	35(44.3%)	27(51.9%)			26(48.1%)	21(55.3%)			9(35.0%)	6(42.9%)		
Yes	44(55.7%)	25(48.1%)			28(51.9%)	17(44.7%)			16(64.0%)	8(57.1%)		
**Margin**			0.43	0.63			0.18	1.81			0.55	0.37
Clear	39(49.4%)	22(42.3%)			29(53.7%)	15(39.5%)			10(40.0%)	7(50.0%)		
Unclear	40(50.6%)	30(57.7%)			25(46.3%)	23(60.5%)			16(60.0%)	7(50.0%)		
**Spiculated margins**			0.25	1.32			0.60	0.27			0.20	1.67
No	7(8.9%)	8(15.4%)			4(7.4%)	4(10.5%)			3(12.0%)	4(28.6%)		
Yes	72(91.1%)	44(84.6%)			50(92.6%)	34(89.5%)			22(88.0%)	10(71.4%)		
**Morphology**			0.25	1.32			0.66	0.20				
Regular	7(8.9%)	8(15.4%)			3(5.6%)	3(7.9%)			4(16.0%)	6(35.7%)		
Irregularly	72(91.1%)	44(84.6%)			51(94.4%)	35(92.1%)			21(84.0%)	9(64.3%)		
**Microcalcifications**											0.86	0.03
No	38(48.1%)	25(48.1%)	0.99	0.02	28(51.9%)	19(50.0%)			10(40.0%)	6(42.9%)		
Yes	41(51.9%)	27(51.9%)			26(48.1%)	19(50.0%)			15(60.0%)	8(57.1%)		
**Adler Flow Grading**			0.03	8.21			0.02	9.87			0.86	0.76
0	12(15.2%)	3(5.8%)			8(14.8%)	2(5.3%)			4(16.0%)	1(7.1%)		
I	32(40.5%)	14(26.9%)			20(37.0%)	6(15.8%)			12(48.0%)	8(57.1%)		
II	24(30.4%)	20(38.5%)			18(33.3%)	17(44.7%)			6(24.0%)	3(21.4%)		
III	11(13.9%)	15(28.8%)			8(14.8%)	13(34.2%)			3(12.0%)	2(14.3%)		
**RI**	0.72±0.07	0.75±0.08	0.04	3.14	0.70±0.11	0.73±0.10	0.04	2.79	0.68±0.11	0.71±0.10	0.07	1.45

Bold formatting is used to highlight the factors or categories in the table and does not indicate any statistical significance or additional meaning.

### 3.2 Construction of clinical feature prediction models

Multivariate logistic regression analysis was performed following a rigorous feature selection process. First, univariate analysis (P<0.1) identified eight potential predictors, including Adler blood flow grading, RI, histological grade, and lymphovascular invasion. To address multicollinearity, variance inflation factors (VIF) were calculated, leading to the exclusion of RI (VIF=6.2) due to its strong correlation with Adler grading. The remaining variables were further refined using LASSO regression with 10-fold cross-validation, ultimately retaining three independent risk factors: lymphovascular invasion (OR 1.82 [95% CI: 1.10–3.49]), maximum lesion diameter (OR 2.28 [95% CI: 1.03–4.34]), and Adler blood flow grading (OR 1.74 [95% CI: 1.17–2.58]). The model was optimized via the Akaike Information Criterion (AIC), with the final selected model demonstrating the lowest AIC value (112.6). Internal validation using 10-fold cross-validation showed stable performance (mean AUC=0.789, 95% CI: 0.730–0.848). The diagnostic performance remained robust in both the training group (AUC=0.807 [95% CI: 0.754–0.860]) and validation group (AUC=0.763 [95% CI: 0.684–0.843]), confirming its generalizability (**Appendix Figure 3**).

### 3.3 Construction and evaluation of radiomics models

Radiomics features were extracted from six groups of ROI regions within the intra-tumoral area and 1-5 mm around the tumor in conventional ultrasound images. A total of 107 radiomics features were extracted from each ROI region, and LASSO regression was used to identify radiomics features closely associated with breast cancer ALNB. Feature weight maps were then drawn (**Appendix Figures 4A, F**). Subsequently, six radiomics models were constructed based on different ROI ranges: intra-tumoral + peri-tumoral 1 mm, intra-tumoral + peri-tumoral 2 mm, intra-tumoral + peri-tumoral 3 mm, intra-tumoral + peri-tumoral 4 mm, and intra-tumoral + peri-tumoral 5 mm (**Appendix Figure 5**). The AUCs of the six models in the training set were 0.650, 0.683, 0.722, 0.788, 0.673, and 0.660, while the AUCs in the validation set were 0.653, 0.699, 0.666, 0.771, 0.671, and 0.657, respectively. Among these, the intra-tumoral + peri-tumoral 3 mm model demonstrated the best diagnostic performance in both the training and validation groups.

By combining the clinical feature model with the optimal intratumoral and peritumoral radiomics model, we developed a clinical-radiomics combined model. The predictive performance of this combined model was evaluated using six different machine learning algorithms (LR, DT, SVM, XGBoost, RF, KNN), and ROC curves with AUC values were generated for both the training and validation cohorts (**Appendix Figures 6A, B**). The results demonstrated that among all the algorithms (**Table 3**), the LR model exhibited the best diagnostic performance, with an Average-AUC of 0.825, Average-Kappa of 0.684, and Average-Accuracy of 0.795, further highlighting the LR model’s strong advantages in clinical applications.

**Table 3:** Evaluation metrics for machine learning algorithms selected using 10-fold cross-validation in the combined predictive model.

Model	Average_ AUC	Average_ Kappa	Average_ Accuracy
Logistic	0.825	0.684	0.795
Tree	0.755	0.523	0.767
SVM	0.722	0.567	0.699
XGBoost	0.712	0.476	0.554
KNN	0.768	0.545	0.673
RF	0.685	0.673	0.737

### 3.4 Comparison of performance among the three predictive models

This study systematically evaluated the performance of the clinical feature model, the optimal intra-tumoral + peri-tumoral radiomics model, and the combined clinical-radiomics model in both the training and validation cohorts. By plotting the ROC curves and calculating the AUC for each model (**Appendix Figures 7A, B**), we found that the combined clinical-radiomics model achieved an AUC of 0.845 (95% CI: 0.795-0.896) in the training group and 0.806 (95% CI: 0.732-0.880) in the validation group, significantly outperforming the models using clinical features alone or radiomics alone. Furthermore, diagnostic performance metrics (**Table 4**) showed that the combined model exhibited the best performance across accuracy, sensitivity, specificity, positive predictive value (PPV), negative predictive value (NPV), Youden index, and F1 score in both the training and validation groups. In the training group, these metrics were 0.897, 0.914, 0.602, 0.904, 0.794, 0.516, and 0.866; in the validation group, they were 0.897, 0.879, 0.663, 0.860, 0.692, 0.542, and 0.835, indicating the strong predictive value of the combined model for AHNB.

**Table 4:** Diagnostic performance of the three predictive model in training and validation groups.

	Accuracy	Sensitivity	Specificity	PPV	NPV	Youden Index	F1 Score
Training							
Model_Cl	0.762	0.859	0.526	0.786	0.628	0.385	0.774
Model_Rad	0.814	0.848	0.617	0.749	0.667	0.465	0.727
Model_Comb	0.897	0.914	0.602	0.904	0.794	0.516	0.866
Validation							
Model_Cl	0.747	0.864	0.511	0.858	0.563	0.375	0.725
Model_Rad	0.787	0.824	0.651	0.786	0.687	0.475	0.793
Model_Comb	0.897	0.879	0.663	0.860	0.692	0.542	0.835

Statistical analysis using DeLong’s test revealed significant differences between the models (**Table 5**). In the training group, the differences between the clinical feature model and the combined model, the optimal intra-tumoral + peri-tumoral radiomics model and the combined model, and the clinical feature model and the optimal radiomics model were statistically significant (P<0.05). In the validation group, the differences between the clinical feature model and the combined model, as well as the radiomics model and the combined model, also reached statistical significance (P<0.05), while no significant difference was observed between the clinical feature model and the optimal radiomics model (P=0.89).

**Table 5:** DeLong's test for three prediction model in training and validation groups.

Training	*P* Value	*P* Value	*P* Value	
Model_Cl	<0.01	0.03		
Model_Rad	<0.01		0.89	
Model_Comb		0.04	<0.01	
	Model_Comb	Model_Rad	Model_Cl	Validation

Bootstrap internal validation of the combined clinical-radiomics model in both the training and validation cohorts yielded the following results: in the training cohort, the calibrated C-index was 0.812 and the Brier score was 0.152, which was better than the threshold of 0.250; in the validation cohort, the calibrated C-index was 0.794 and the Brier score was 0.214, also below the threshold of 0.250. The calibration curves in both cohorts closely aligned with the ideal curve, demonstrating the high predictive accuracy and calibration of the model, which supports its reliability and effectiveness in clinical applications (**Appendix Figures 8A, B**).

Decision curve analysis (DCA) results (**Appendix Figures 9A, B**) indicated that the combined clinical-radiomics model provided significant net benefits over a wide range of risk thresholds (10%-90% in the training group, and 20%-65% in the validation group), outperforming both “treat-all” and “treat-none” strategies. Notably, at the threshold of 52%, the model achieved an optimal balance between sensitivity and specificity, enhancing the identification of AHNB patients without increasing the risk of misdiagnosis. These findings suggest that the model holds strong clinical utility and potential decision-support value for predicting AHNB, aiding in the optimization of patient management and minimizing unnecessary interventions.

### 3.5 Visualization analysis of the predictive model

To further enhance the clinical applicability of the combined clinical-radiomics predictive model, we visualized the model and constructed a nomogram for predicting AHNB risk (**Appendix Figure 10**). The nomogram incorporated several key variables, including histological grade, vascular invasion, Adler vascular grade, and Rad Score. Each variable was assigned a weighted score based on its relative importance in predicting AHNB. Among these, the Rad Score was identified as an independent risk factor for AHNB in multivariate analysis, with a score range from -0.1 to 1 point, corresponding to a weighted score of 0 to 100 in the nomogram. The length of the Rad Score line in the nomogram indicates its substantial impact on predicting AHNB compared to other variables.

In practice, clinicians can calculate a total score by summing the individual scores of each variable and quickly determine the predicted probability of AHNB using the “Probability” axis at the bottom of the nomogram. For instance, in the training group, a patient with vascular invasion, a maximum tumor diameter > 2 cm, an Adler vascular grade of II, and a Rad Score of 0.344 would have a total score of 62 points, corresponding to a 72% probability of AHNB. This visualization tool not only enhances the model’s clinical usability but also provides clinicians with an intuitive and quantitative risk assessment method, facilitating personalized treatment decision-making.

## 4 Discussion

The status of ALN is a critical prognostic factor in breast cancer, as it plays a key role in determining the pathological staging of the disease (16). ALND is the standard procedure used to assess ALN status, accurately stage the disease, and remove potentially metastatic lymph nodes. However, ALND can lead to severe complications, such as shoulder dysfunction and arm lymphedema, which negatively affect the quality of life (17). Therefore, minimizing unnecessary ALND has become an urgent issue. Studies (18) have shown that for patients with ≤2 metastatic axillary lymph nodes (ALNM, low ALN metastatic load), there is no statistically significant difference in local recurrence rates and 10-year overall survival between the ALND and non-ALND groups. However, patients with more than 2 ALNM (high ALN metastatic load) face a higher risk of local recurrence and are thus better suited for neoadjuvant chemotherapy or ALND (19). Therefore, accurately predicting ALNB before ALND is crucial for determining appropriate treatment plans. In this study, we developed a predictive model for AHNB by analyzing the clinical ultrasound and radiomics characteristics of the primary tumor and ALNB, creating a visual nomogram to assist in individualized preoperative assessment and treatment planning, ultimately supporting better clinical decision-making.

This study included 131 patients with ALNM. Univariate and multivariate analyses identified vascular invasion, maximum tumor diameter, and Adler blood flow grading as independent risk factors for AHNB. Metastasis in malignant tumors is a complex process involving multiple steps, including tumor cell detachment, invasion, migration, entry into the vascular system, evasion of immune responses, infiltration of new sites, and growth. Among these, tumor vascular invasion is a key step. Research shows that interactions among tumor cells, stromal cells, and lymphatic endothelial cells via growth factors, cell surface receptors, and cytokines form a complex regulatory network. For instance, CXCL12 and SLC (CCL-21) bind to CXCR4 and CCR7 receptors on breast cancer cells, respectively, promoting tumor cell migration to the lymphatic system along a chemotactic gradient (20). Our findings indicate that although vascular invasion was not an independent risk factor for ALNM, it was significantly correlated with AHNB, with patients exhibiting vascular invasion being 1.82 times more likely to develop AHNB compared to those without vascular invasion, which is consistent with the findings of Luo et al. (21).

The selection of the 2 cm threshold for distinguishing T1 and T2 tumors in this study was grounded in both the AJCC TNM staging criteria and its biological relevance to metastatic potential (22). While the AJCC system defines T1 tumors as ≤2 cm and T2 tumors as >2 cm, our rationale extended beyond staging conventions. Receiver operating characteristic (ROC) curve analysis confirmed that 2 cm optimally balanced sensitivity (78%) and specificity (64%) for predicting AHNB (AUC=0.71, 95% CI: 0.63–0.79; Youden index=0.42). Furthermore, continuous tumor diameter analysis revealed a dose-dependent relationship, with each 1 cm increase in size associated with a 1.41-fold elevated AHNB risk (95% CI: 1.12–1.78, p=0.03). Biologically, tumors exceeding 2 cm exhibit heightened invasiveness, as demonstrated by Dihge et al. (23) suggested that larger tumors tend to be more aggressive, breaking through breast tissue more easily and spreading to nearby lymph nodes, significantly increasing the risk of ALNM. Moreover, larger tumors are often associated with increased angiogenesis, providing additional nutrients and oxygen while facilitating distant metastasis via lymphatic or blood routes. Tumor growth also leads to significant changes in the local microenvironment, such as immune suppression or activation of pro-metastatic pathways, which allow tumor cells to thrive in lymph nodes and increase the likelihood of ALNM. These factors collectively explain why patients with tumors >2 cm more prone to AHNB. However, some studies have reported (24, 25) no clear relationship between tumor size and ALNB, possibly due to other factors such as tumor molecular subtype, cell proliferation rate, and immune responses, which also influence metastatic potential. Therefore, the relationship between tumor size and ALNB requires further investigation.

Our results showed a significant correlation between Adler blood flow grading (II-III) and AHNB. The number of blood vessels in breast cancer plays a crucial role in tumor growth, invasion, and metastasis. During tumor growth, factors such as hypoxia and activation of oncogenes can induce the expression of vascular endothelial growth factor (VEGF), promoting angiogenesis within the tumor and facilitating metastasis through the vascular network, thereby increasing tumor invasiveness. While intratumoral vascularization does not directly affect surrounding lymphatic vessels, it provides nutrients necessary for tumor growth and invasion, increasing the risk of ALNM. Studies have also demonstrated a positive correlation between angiogenic factors and tumor growth and metastasis, with microvascular blood flow detection serving as an important prognostic indicator in breast cancer. VEGF is particularly significant in predicting breast cancer prognosis. However, findings by Yi et al. (26) contradict our results, suggesting no significant correlation between Adler blood flow grading and AHNB. The discrepancy may stem from differences in case distribution and intergroup discriminative power of Adler grading. In Yi et al.’s study, the proportions of Adler II-III grades in the LNB and LHB groups were 79% and 81%, respectively (p=0.84), rendering this metric ineffective for distinguishing the two groups. In contrast, our study demonstrated a significantly higher proportion of Adler II-III grades in the LHB group compared to the LNB group (p<0.05). This discrepancy may be explained by the fact that while tumor vasculature acts as a transport medium for tumor cells, it does not directly affect lymphatic function. The spread, deposition, and proliferation of cancer cells in draining lymph nodes depend on their quantity and invasive growth patterns, which could account for the lack of significant association between Adler blood flow grading and AHNB in some studies.

Some studies have suggested that (27) pathological subtypes are prognostic factors in breast cancer, but our study found no significant statistical difference, possibly because there is no linear correlation between pathological subtypes and malignancy. Additionally, our study categorized tumor burden based on ALNM, which might have contributed to the lack of differentiation in pathological subtypes. With the advancement of radiomics, it has become an important tool for breast cancer diagnosis and prognosis. Radiomics, by extracting large quantities of quantitative features from medical images, captures subtle differences not detectable through conventional imaging, providing new evidence for clinical decision-making. By analyzing intratumoral and peritumoral radiomic features, our study identified significant features associated with AHNB, and combining intratumoral and peritumoral features significantly improved prediction accuracy.

The analysis of radiomic features involved a rigorous multi-step workflow to ensure robustness and biological relevance. First, 107 radiomic features were extracted from both intratumoral and peritumoral (1–5 mm) regions using standardized protocols, including shape-based descriptors, first-order statistics, gray-level co-occurrence matrix (GLCM) textures, and wavelet-transformed features. To address reproducibility, features with intraclass correlation coefficients (ICC) <0.8 across two independent observers were excluded, retaining 30 stable features. Subsequent dimensionality reduction employed Maximum Relevance Minimum Redundancy (MRMR) to prioritize high-discriminative, low-redundancy features, followed by LASSO regression with 10-fold cross-validation, ultimately identifying five key predictors (e.g., wavelet-HLH_GLCM_Correlation). These wavelet-based features capture heterogeneity at the tumor-stroma interface, potentially reflecting spatial patterns of lymphatic invasion.

When evaluating different peritumoral regions, the intratumoral + peritumoral 3 mm model achieved the highest AUC (training: 0.722; validation: 0.771), outperforming narrower (1–2 mm) and broader (4–5 mm) regions. This suggests that the 3 mm peritumoral zone contains microenvironmental cues—such as immune cell infiltration or angiogenesis—critical to lymph node metastasis, while wider regions introduce noise from normal tissue. The decline in performance beyond 3 mm aligns with Dong et al.’s findings (28), which emphasize the tumor microenvironment’s spatially constrained role in metastatic behavior. These results underscore the importance of balancing biological relevance and computational complexity when defining radiomics regions of interest (ROIs).

Based on the ultrasound radiomics model, we further developed a combined model incorporating clinical and radiomic features. The combined model outperformed both the clinical feature model and the radiomics model alone in terms of AUC, ACC, and other diagnostic metrics in both training and validation cohorts. This confirms that integrating clinical and radiomic features enhances the capture of multidimensional information and improves AHNB prediction. The combined model built using LR demonstrated superior diagnostic performance compared to other machine learning algorithms, confirming the advantages of LR models in clinical applications. This model not only achieved high diagnostic performance but also exhibited good generalizability, helping clinicians more accurately assess lymph node status in real-world practice.

Additionally, we validated the model’s consistency and clinical utility through multiple evaluation methods. Bootstrap analysis showed that the calibration curves for both the training and validation cohorts exhibited high accuracy, indicating the model’s stable and accurate AHNB risk prediction. Moreover, decision curve analysis (DCA) demonstrated significant net benefit across a wide range of risk thresholds, particularly at the 52% cutoff, where the model effectively balanced sensitivity and specificity. This suggests that the model can aid clinicians in making more precise treatment decisions, reducing unnecessary interventions and enhancing individualized patient management.

While several studies have shown that radiomics-based models have good predictive value for breast cancer AHNB, limitations still exist (13, 29–31). Wu et al. (13) explored a prediction model for ALNB in breast cancer patients with ALNM, combining ultrasound radiomics and clinical features. The results in their training and validation cohorts were 0.816 and 0.577, respectively, indicating limitations such as overfitting and the exclusion of peritumoral radiomic features. In contrast, our study addresses these limitations by incorporating both intratumoral and peritumoral radiomic features along with clinical characteristics, thus enhancing AHNB prediction.

Lu et al. (31) developed an ALNB prediction model for breast cancer using preoperative MRI-based radiomic features, visualized with a nomogram, with an AUC of only 0.79. Their study was limited by the use of a single dataset and reliance on SVM, a nonlinear classification algorithm that is not well-suited for constructing nomograms, which are typically based on linear or interpretable models such as LR. In comparison, our study employed multiple classifiers for the combined prediction model, using LR for the nomogram, and validated the results with a test set, ensuring greater reliability.

Despite these contributions, this study has some limitations. First, the sample size is relatively small, and future research should expand the cohort to verify the model’s generalizability. Second, the extraction and selection of radiomic features depend on existing algorithms and parameter settings, which may affect the model’s robustness. Future research should focus on developing standardized radiomic workflows to enhance comparability across studies. Third, The exclusion of tumors >5 cm and multifocal lesions aimed to focus on predicting lymph node burden in early-stage breast cancer (T1–T2). However, this may limit the model’s applicability to advanced-stage or multifocal patients. Finally, while this study focused on preoperative ultrasound data, incorporating additional imaging modalities, such as MRI or CT, could further improve the model’s accuracy and provide a more comprehensive assessment of ALNB.

## 5 Conclusion

This study developed a noninvasive predictive model for AHNB in breast cancer patients, combining clinical and ultrasound radiomic features. The model demonstrated high diagnostic performance and clinical utility, providing a valuable tool for individualized treatment planning and decision-making in breast cancer management. By integrating intratumoral and peritumoral radiomic features, the model captures multidimensional information that improves the accuracy of AHNB prediction. Future studies should focus on validating the model in larger, multicenter cohorts and exploring the potential of incorporating additional imaging modalities to further enhance prediction accuracy.

## Data Availability

The raw data supporting the conclusions of this article will be made available by the authors, without undue reservation.
