# Supplementary material for: Machine learning-driven ultrasound radiomics for assessing axillary lymph node burden in breast cancer
Source: Front Endocrinol (Lausanne). 2025 Feb 27;16:1548888. doi: 10.3389/fendo.2025.1548888 (PMC11903272; doi:10.3389/fendo.2025.1548888)

Appendix Figure 1-1. Depicts the schematic of the intertumoral region of interest.


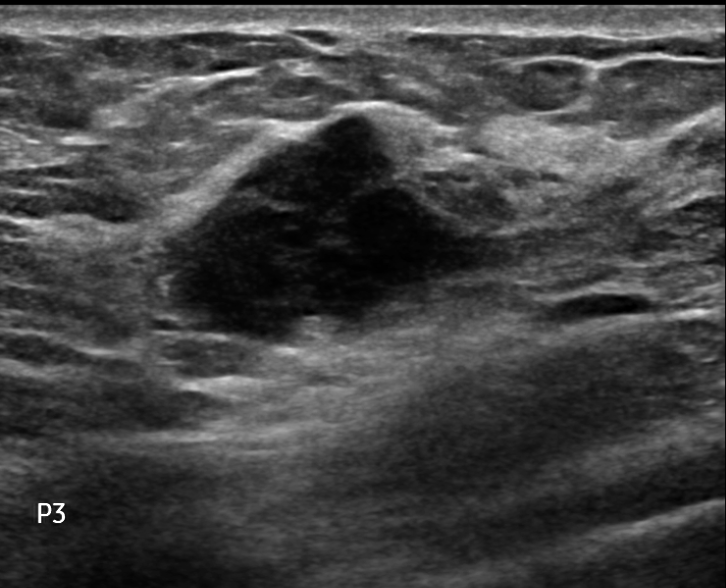

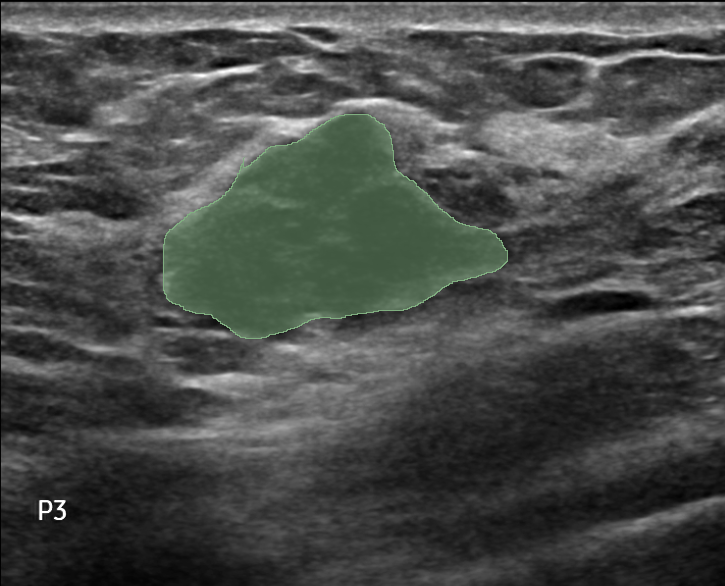


Appendix Figure 1-2. Depicts the schematic of the peritumoral region of interest.


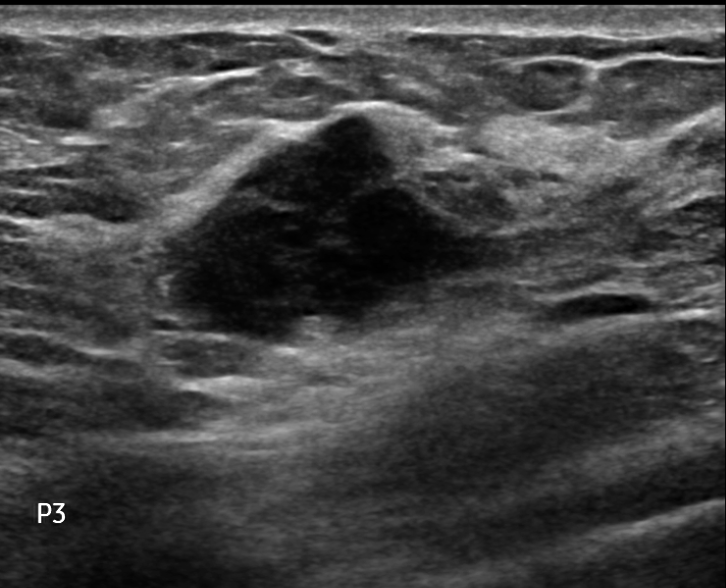

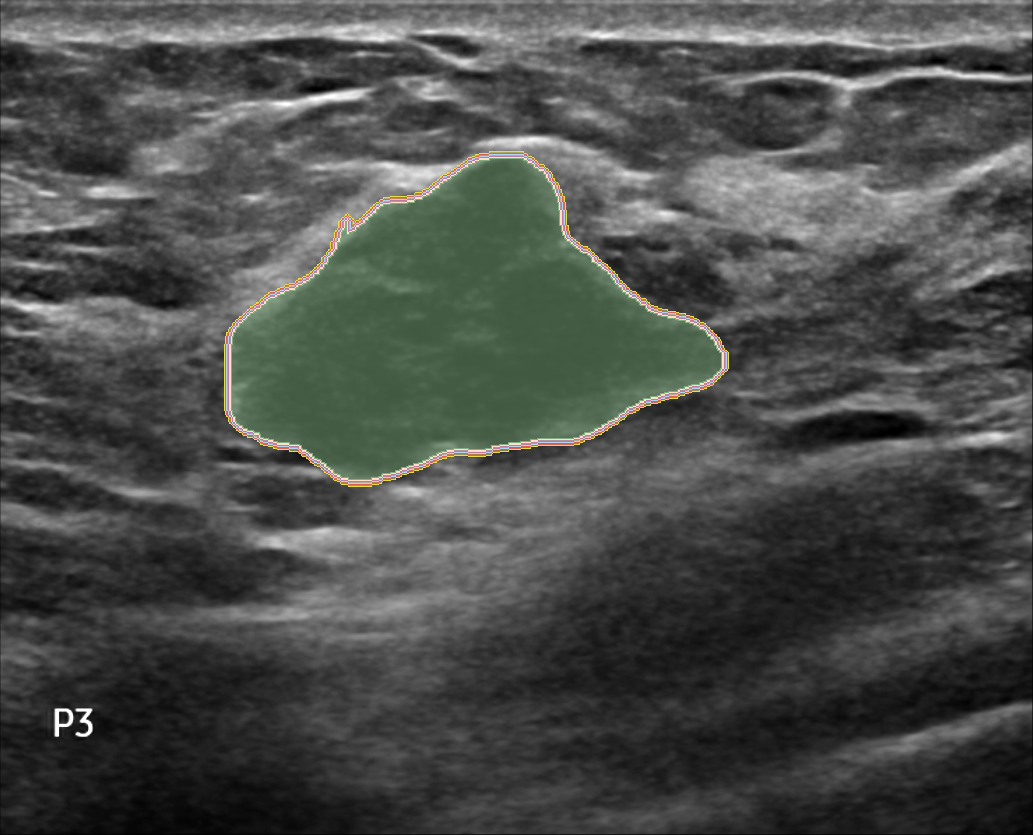


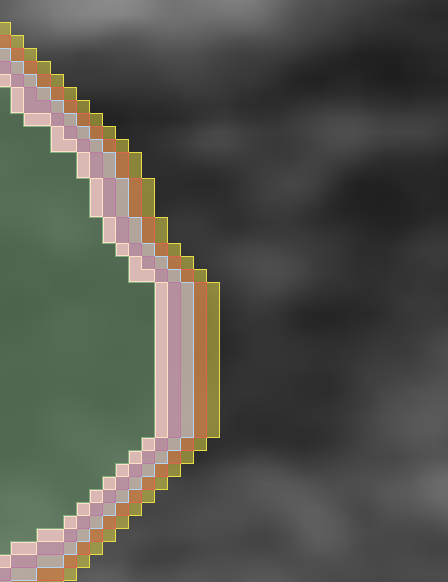


Appendix Figure 2**.** Correlation analysis of clinical features.


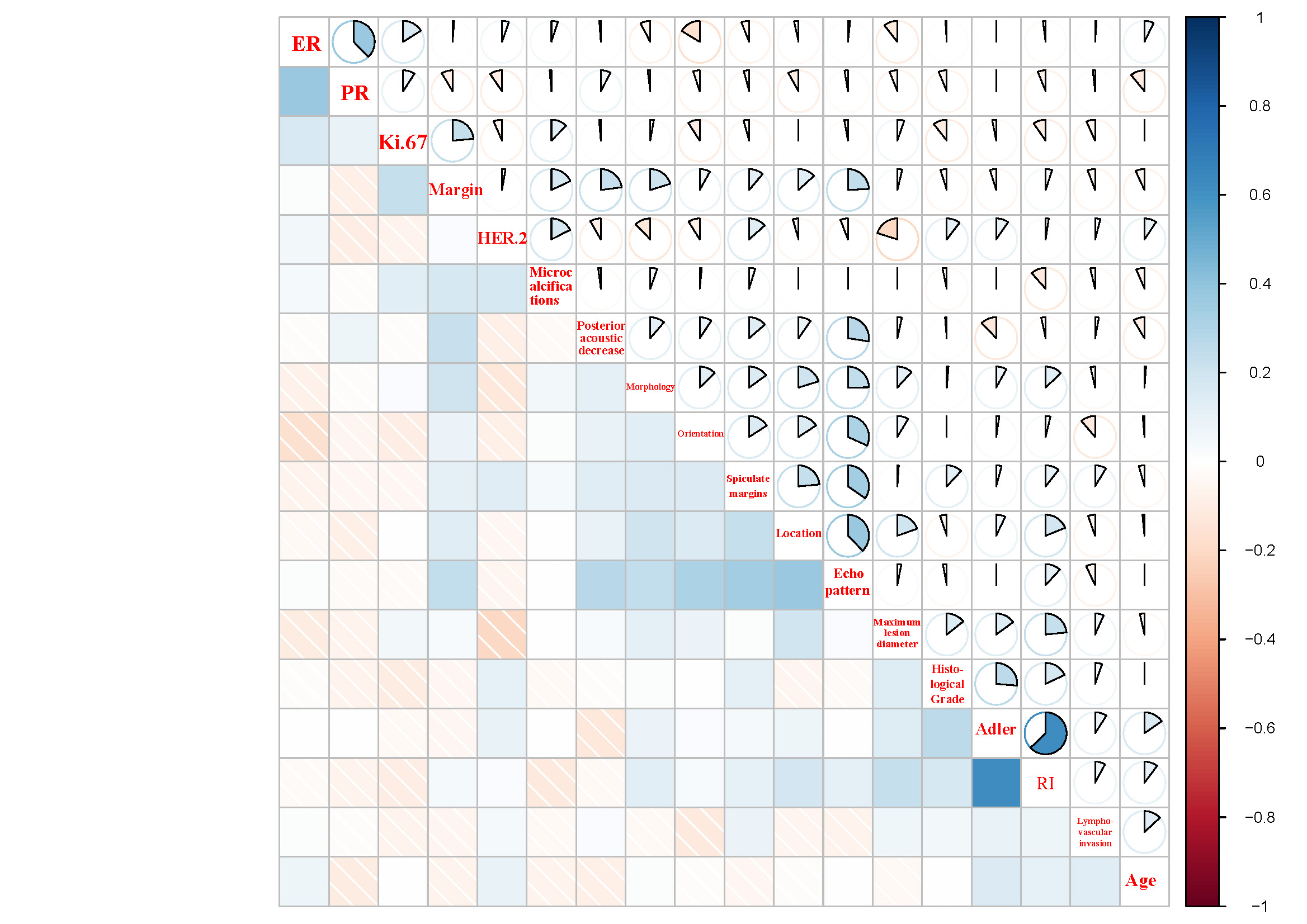


Appendix Figure 3. Multivariate Analysis of Ultrasound and Clinicopathological Features in Predicting ALNB in Breast Cancer


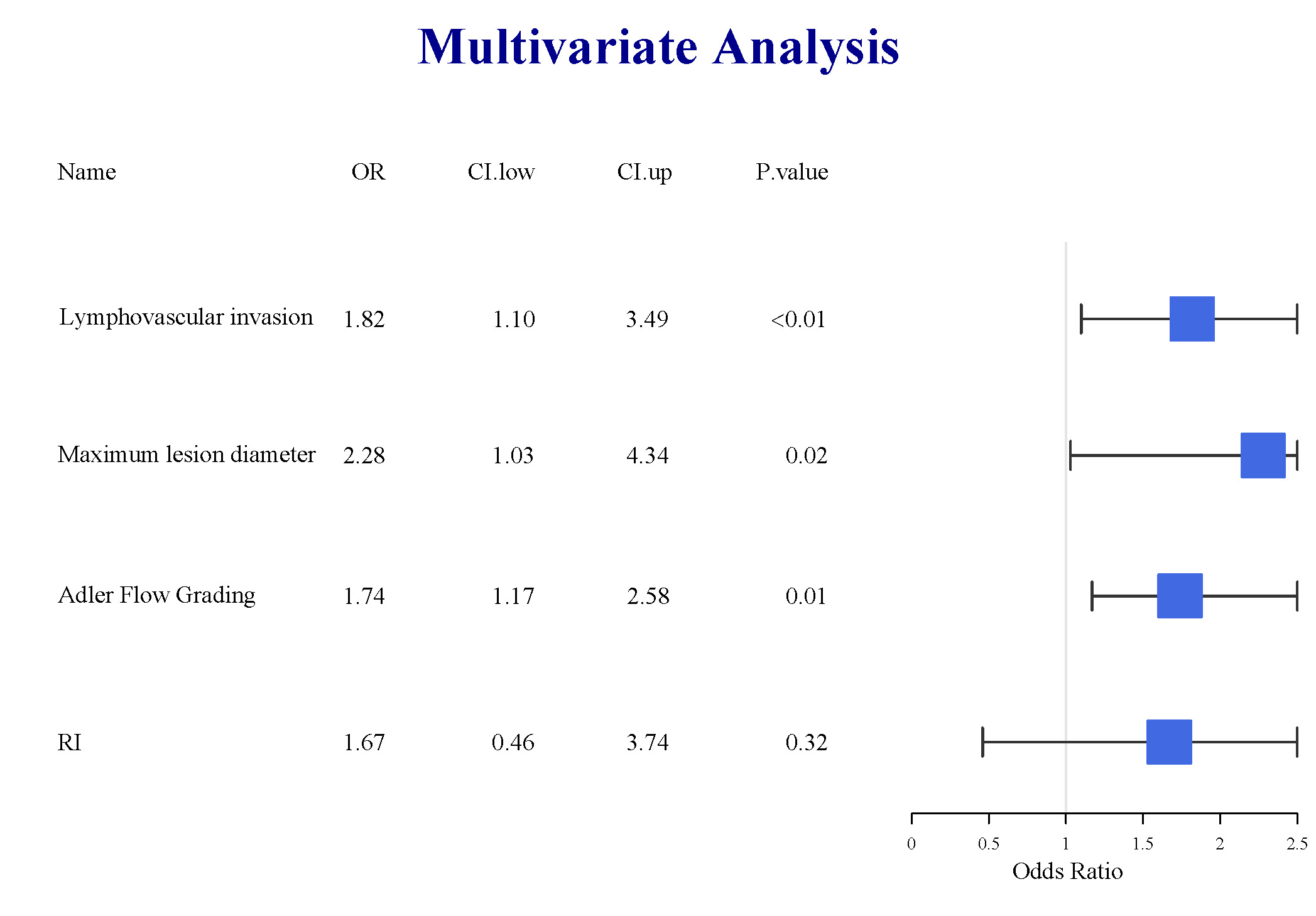


Appendix Figure 4. Radiomics Feature Selection Using LASSO Logistic Regression

(a: Intratumoral Features; b: Intratumoral + Peritumoral 1 mm Features; c: Intratumoral + Peritumoral 2 mm Features; d: Intratumoral + Peritumoral 3 mm Features; e: Intratumoral + Peritumoral 4 mm Features; f: Intratumoral + Peritumoral 5 mm Features)


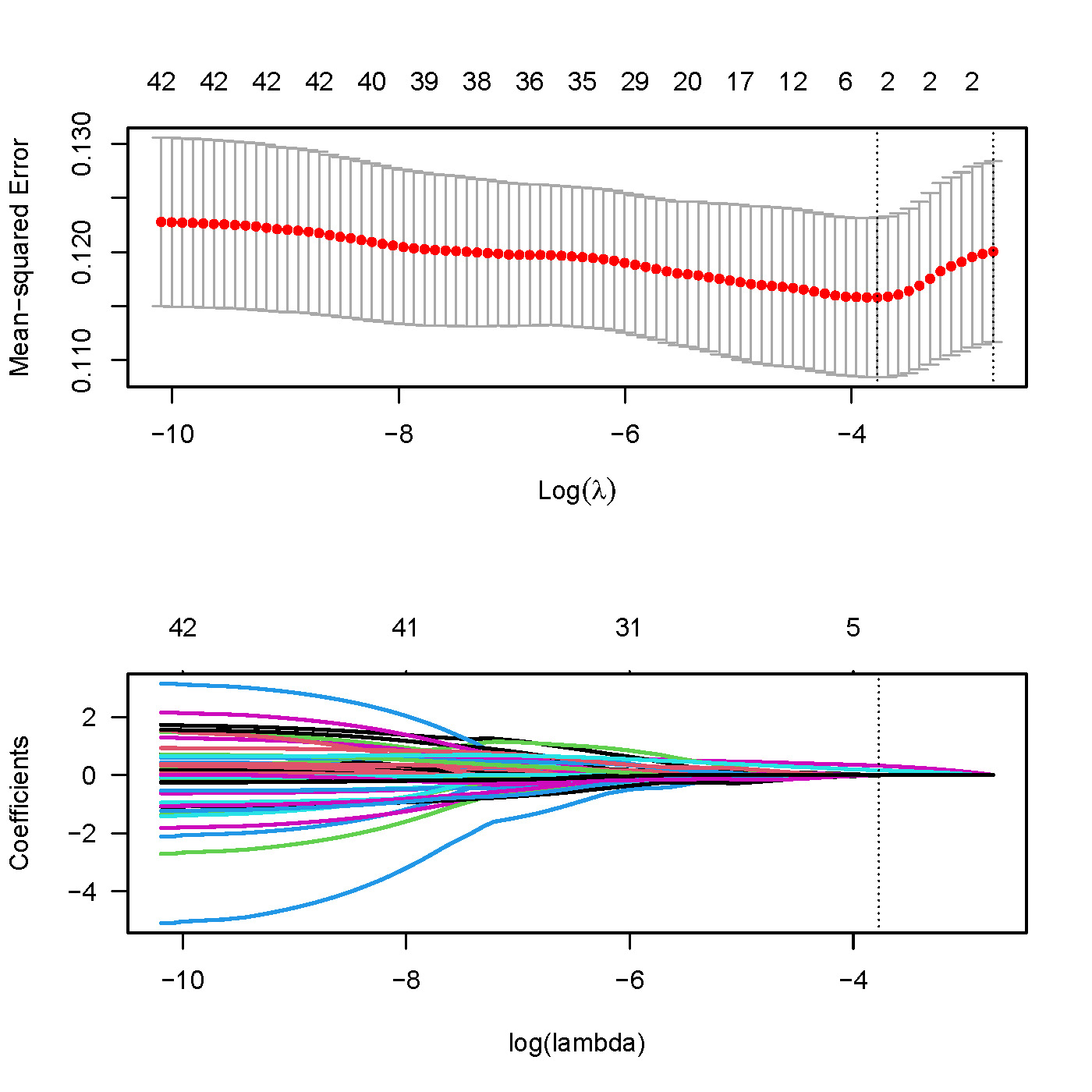

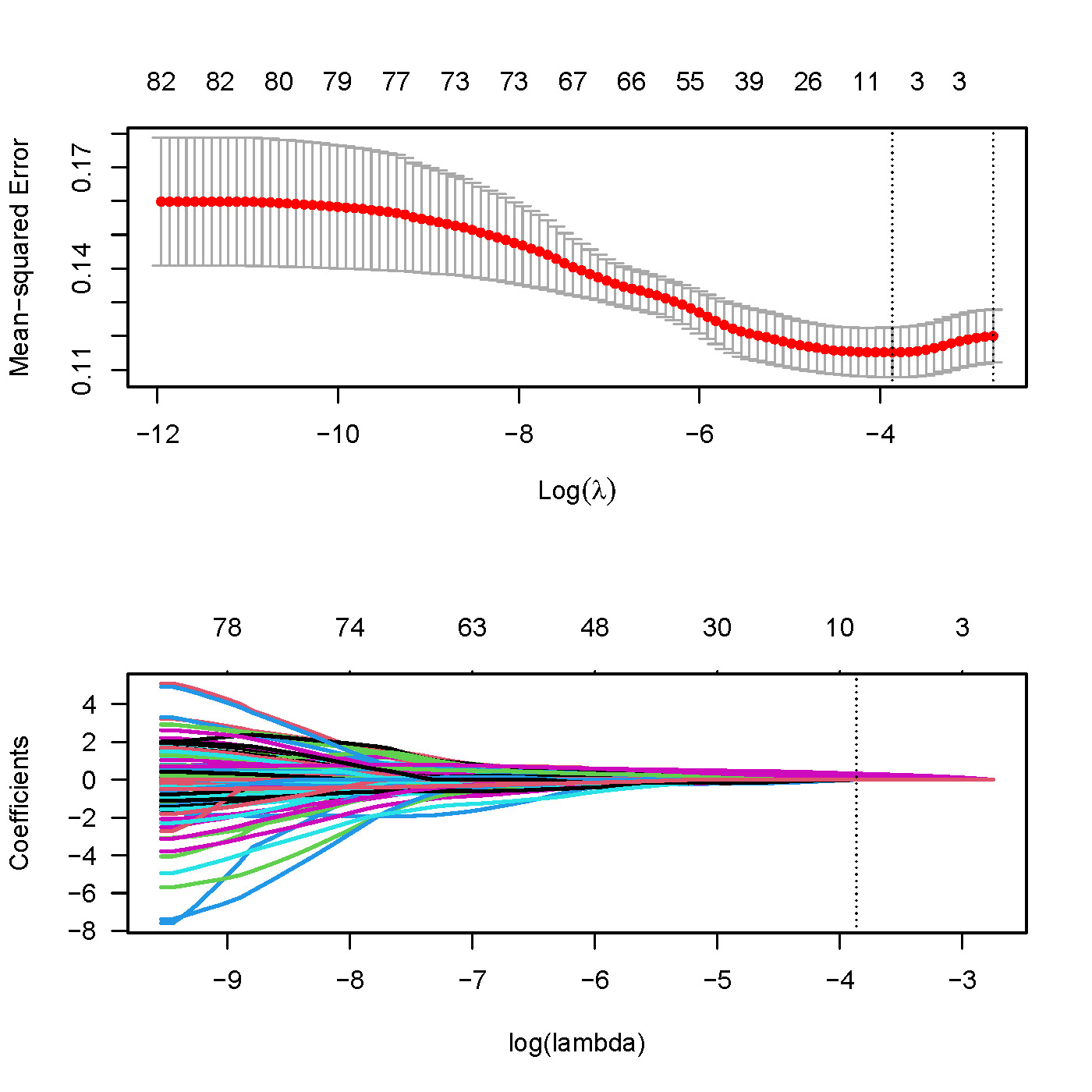

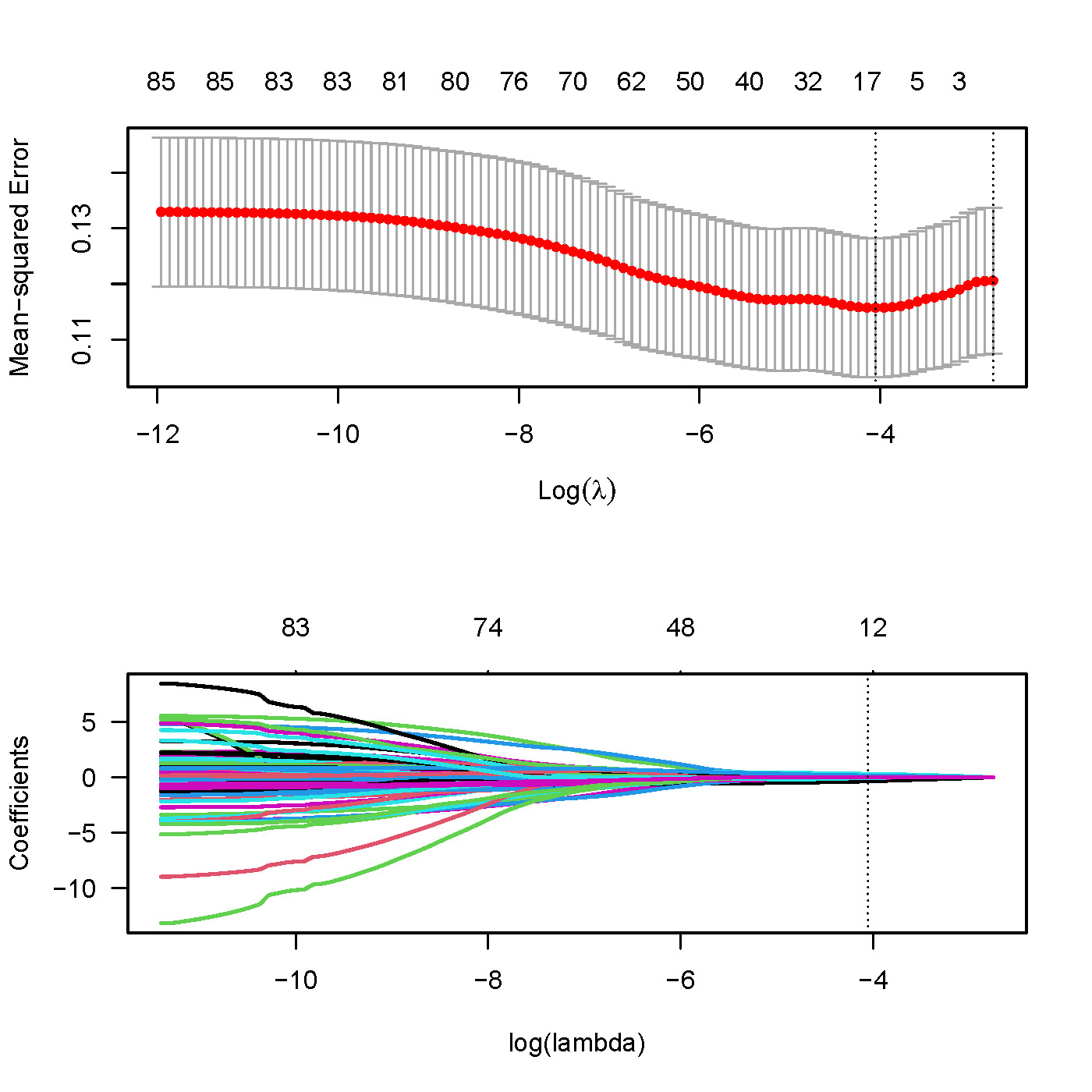


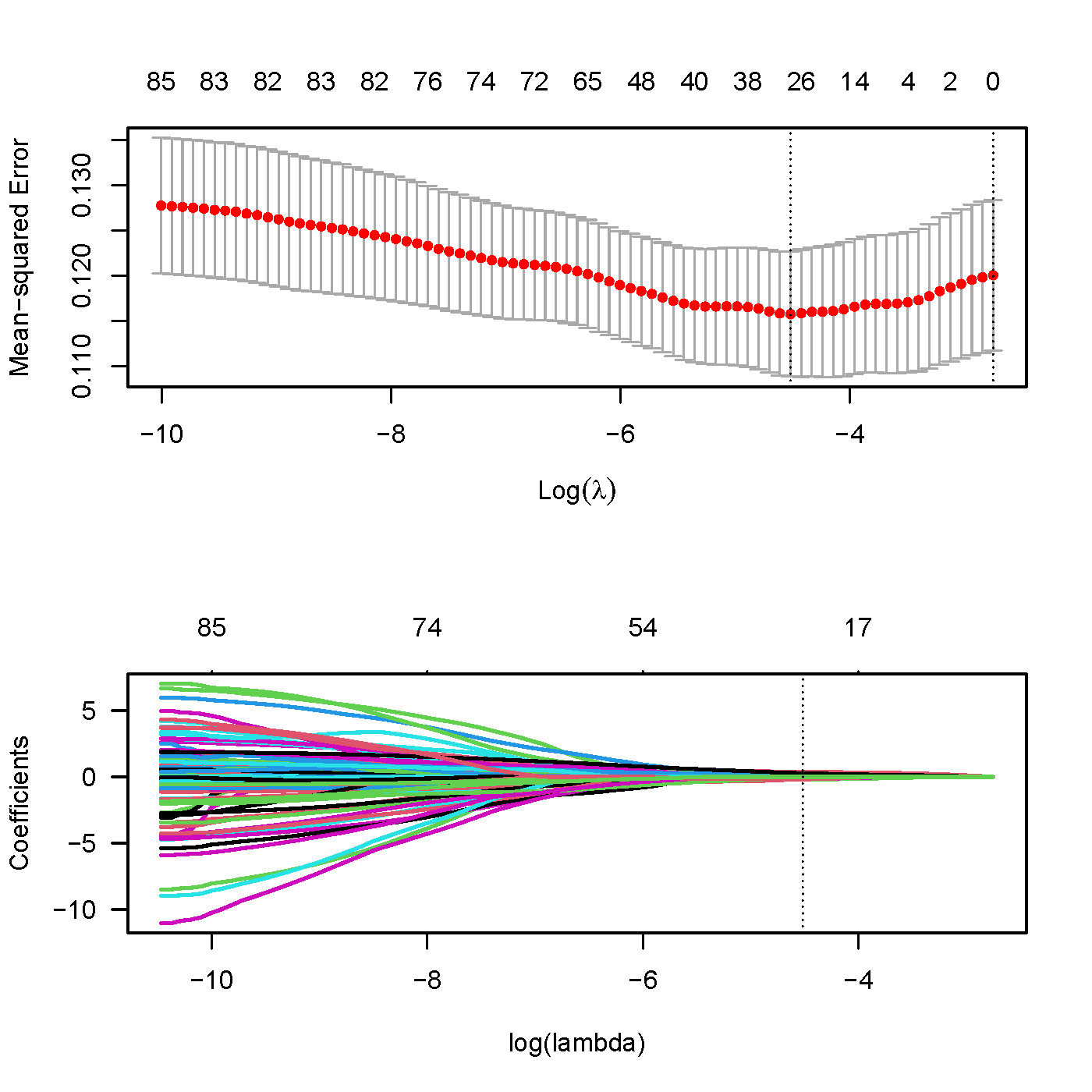

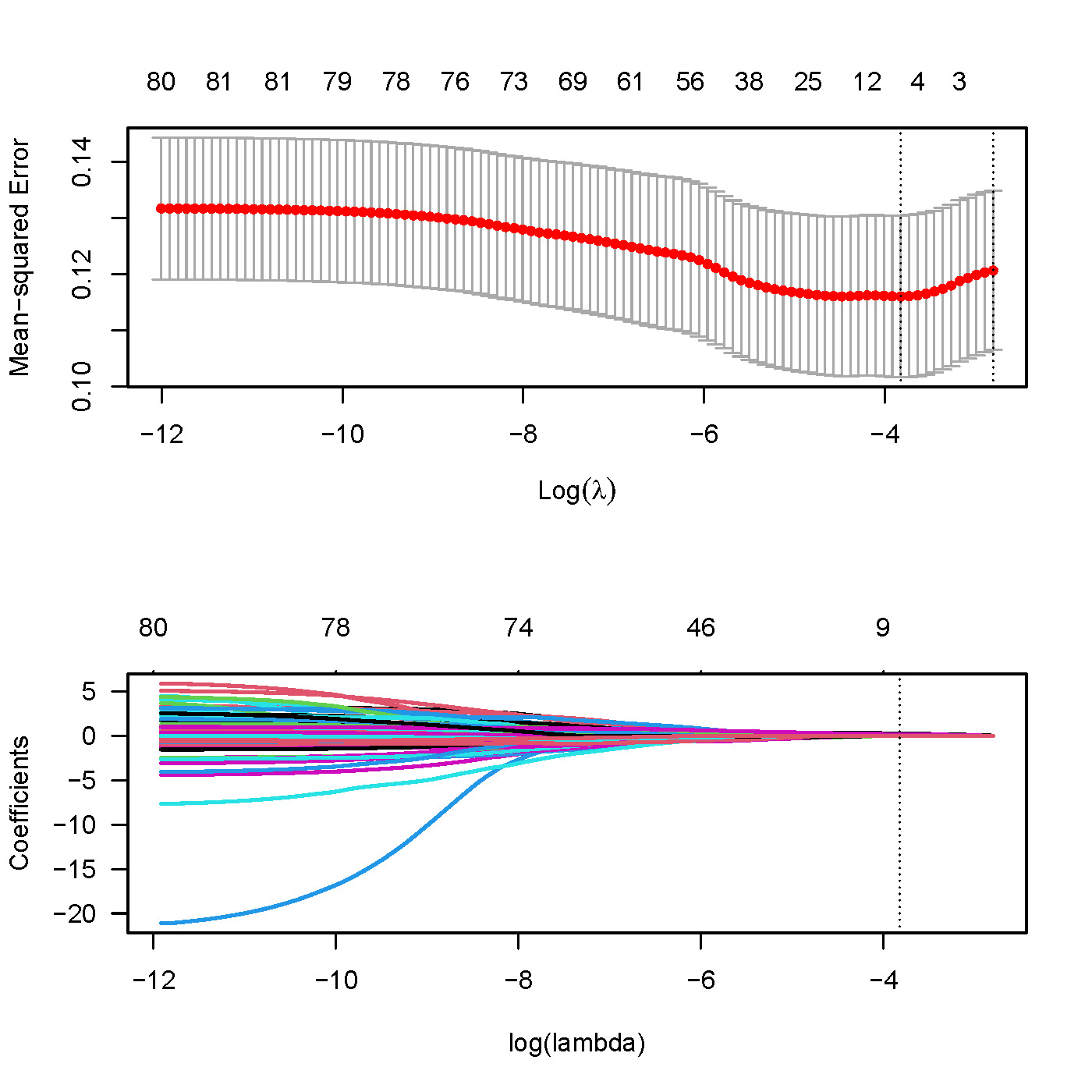

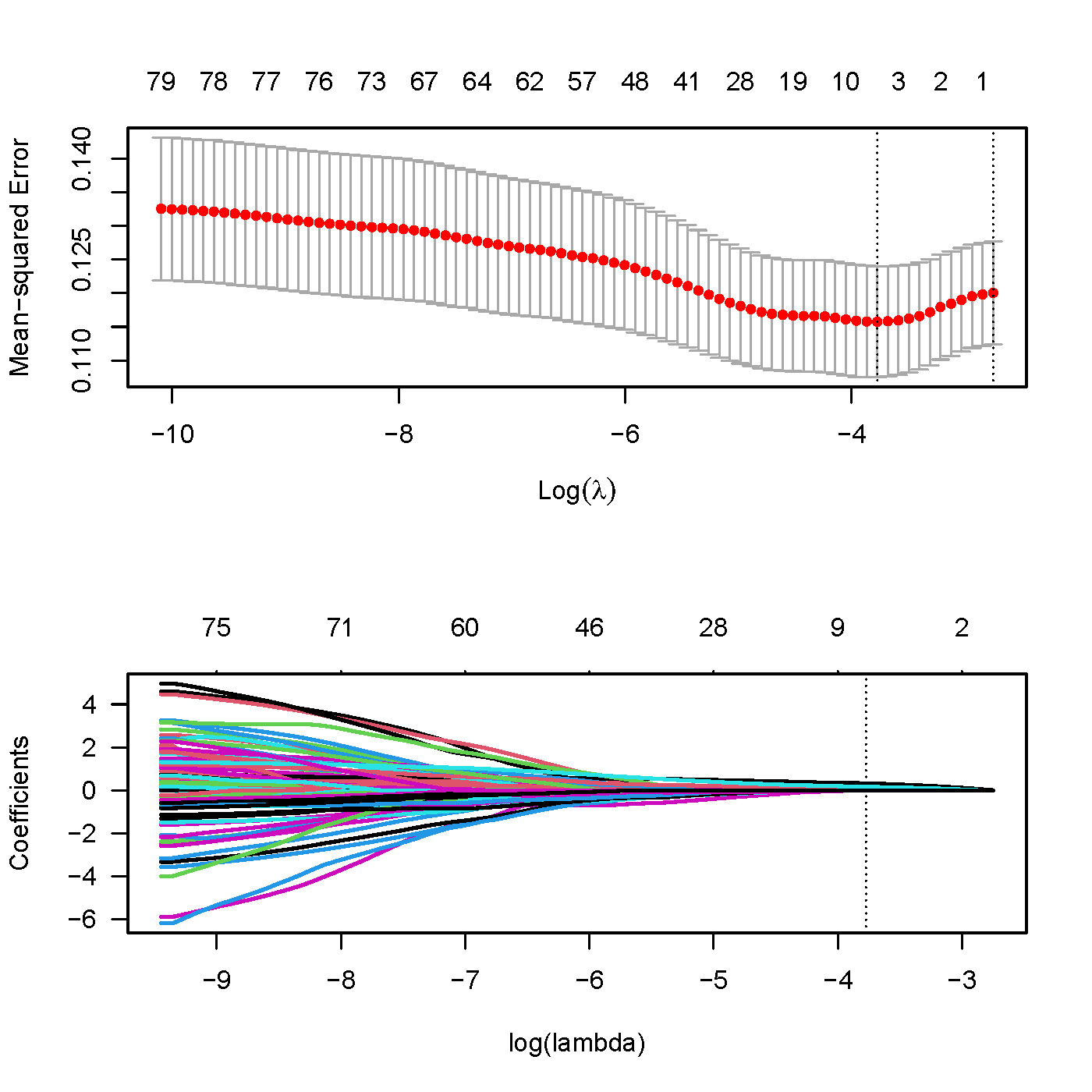


Appendix Figure 5. ROC Curves of Intratumoral and Peritumoral 1~5 mm Radiomics Prediction Models in Training and Validation Sets


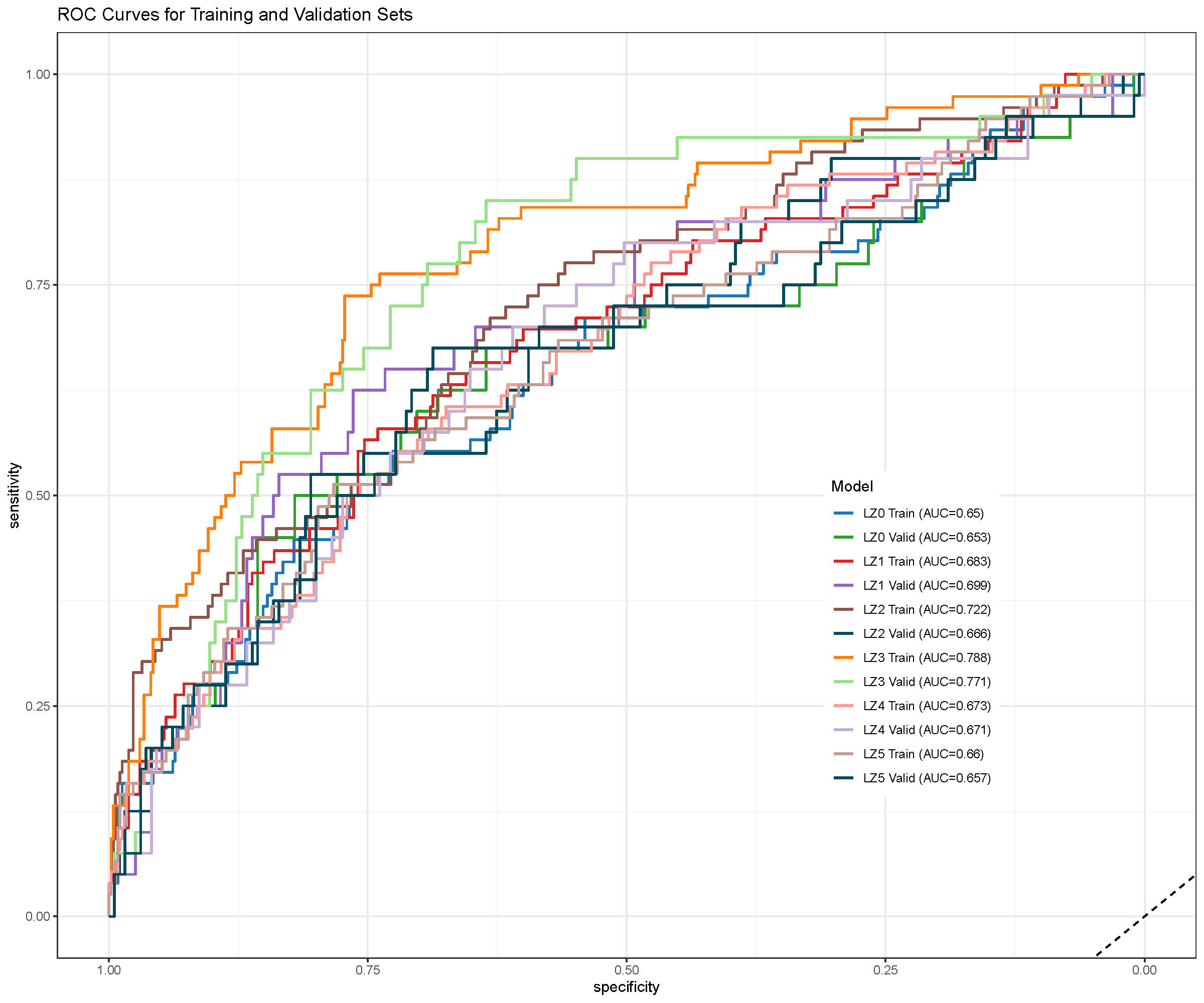


Appendix Figure 6. ROC Curves of Combined Prediction Models Based on Six Machine Learning Methods (a: Training Set; b: Validation Set)


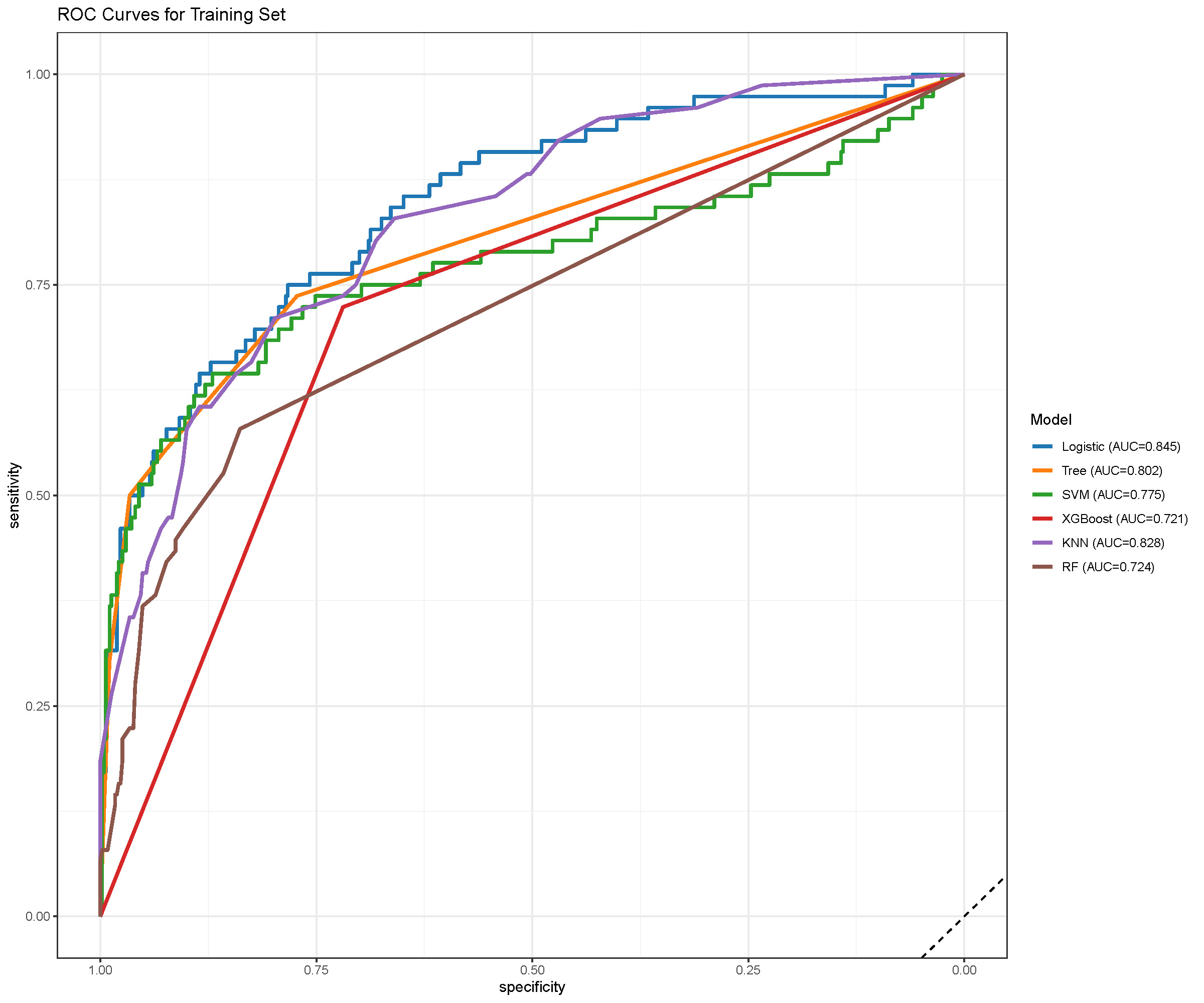

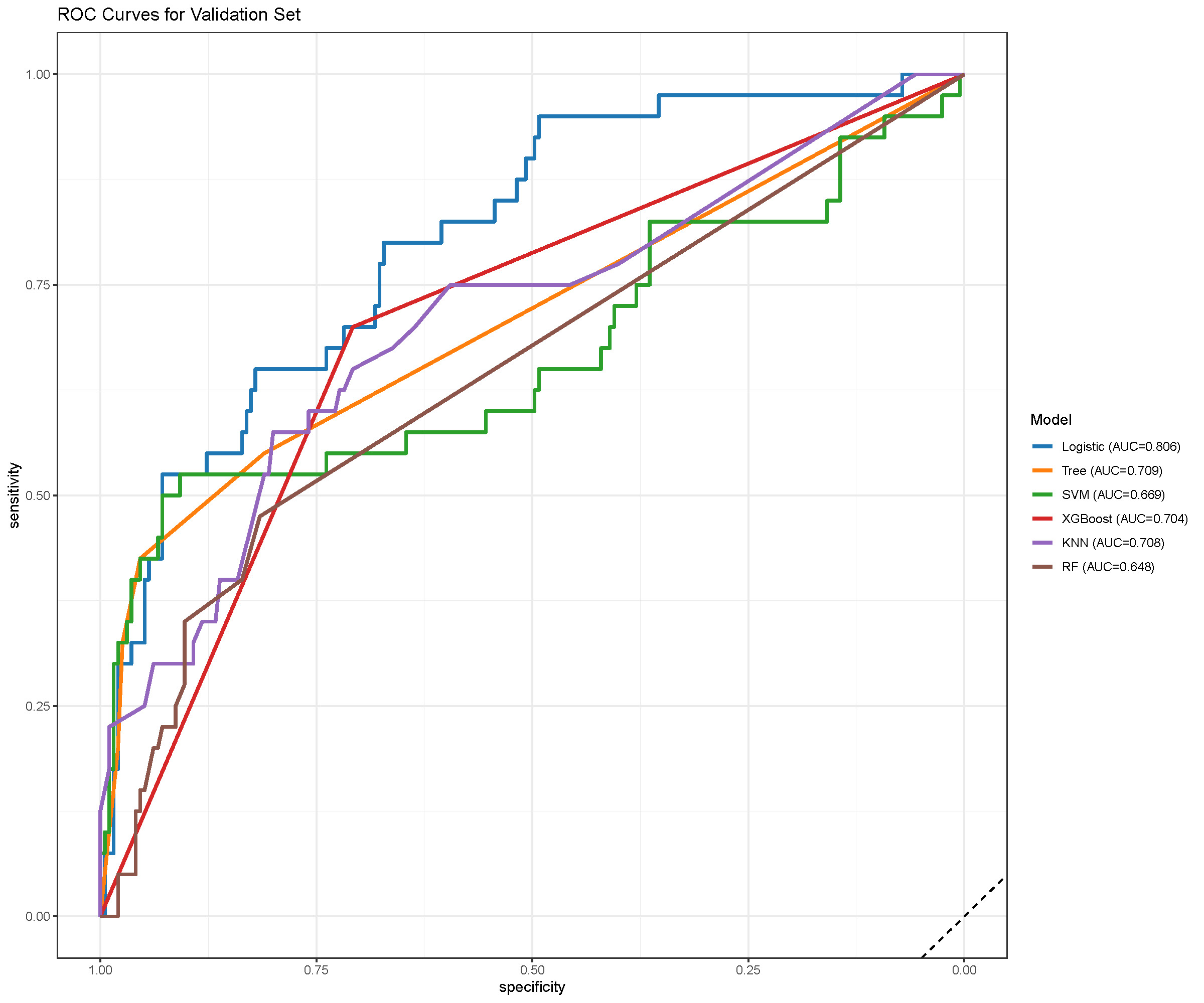


（a） （b）

Appendix Figure 7. ROC Curves of Three Prediction Models in Training and Validation Sets

(a: Training Set; b: Validation Set)


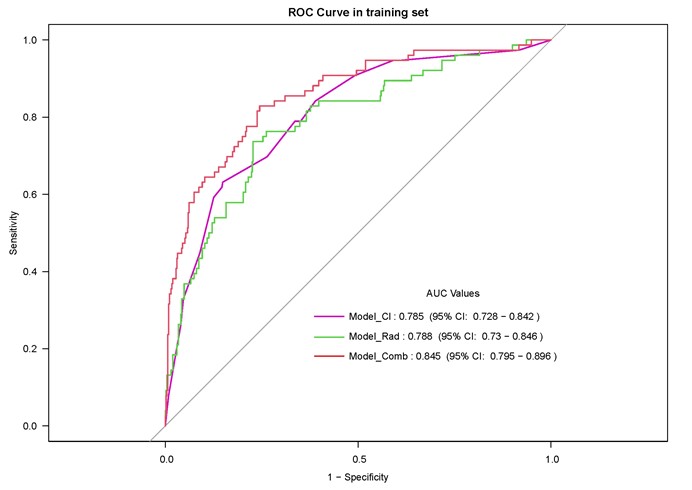

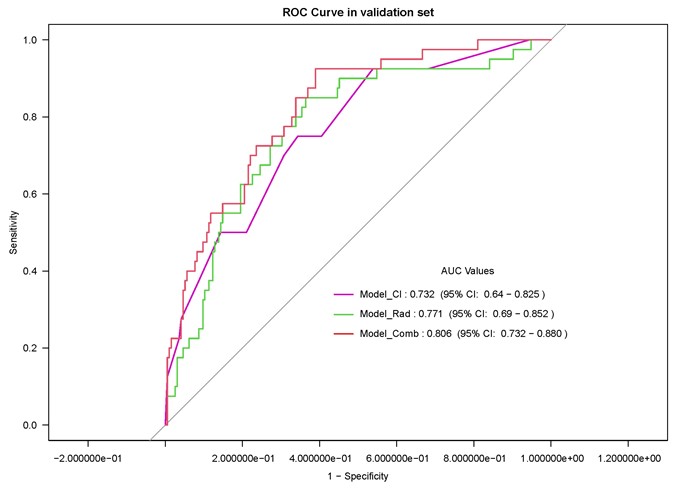


（a） （b）

Appendix Figure 8. Calibration Curves of Nomogram for Predicting ALNB in Breast Cancer Patients

(a: Training Set; b: Validation Set)


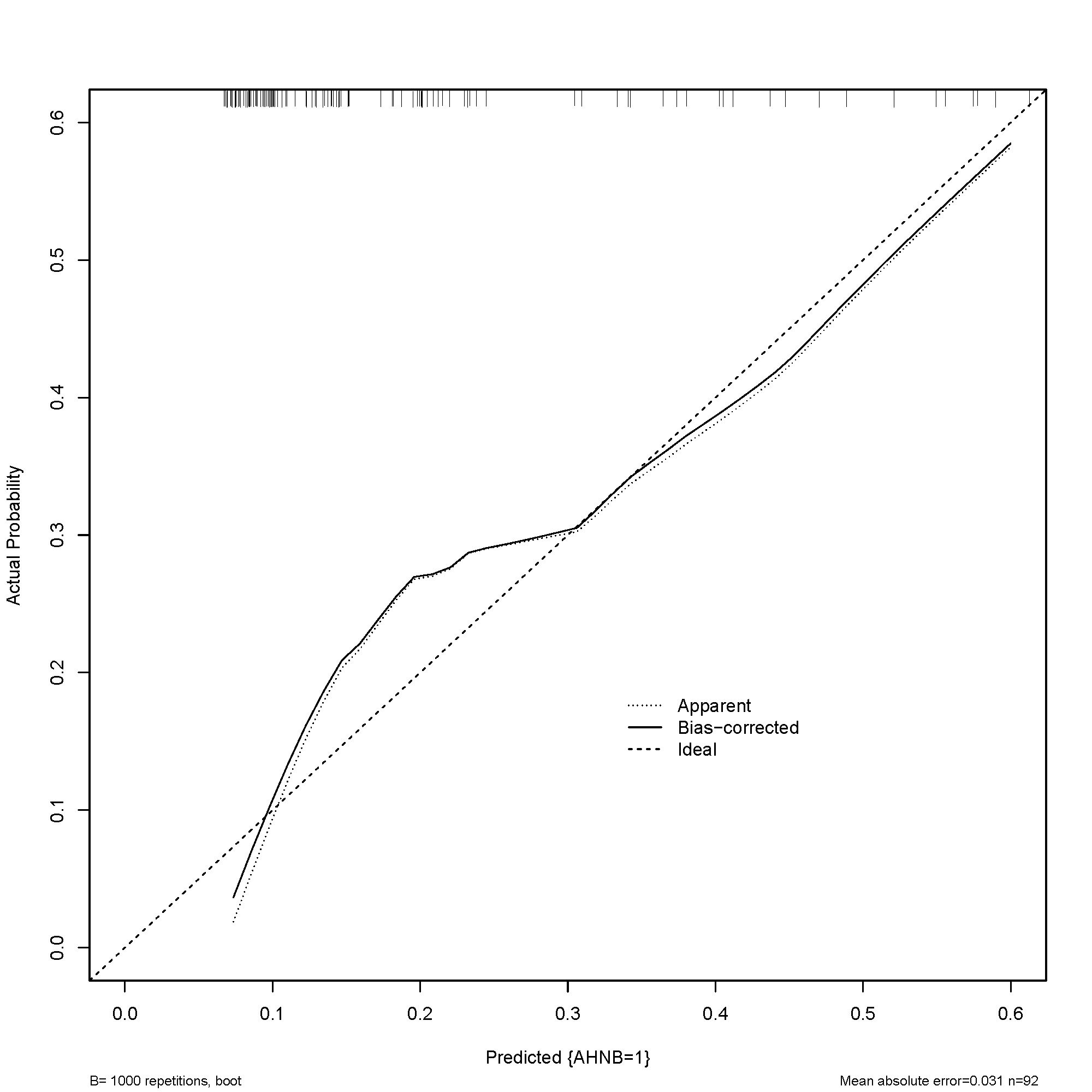

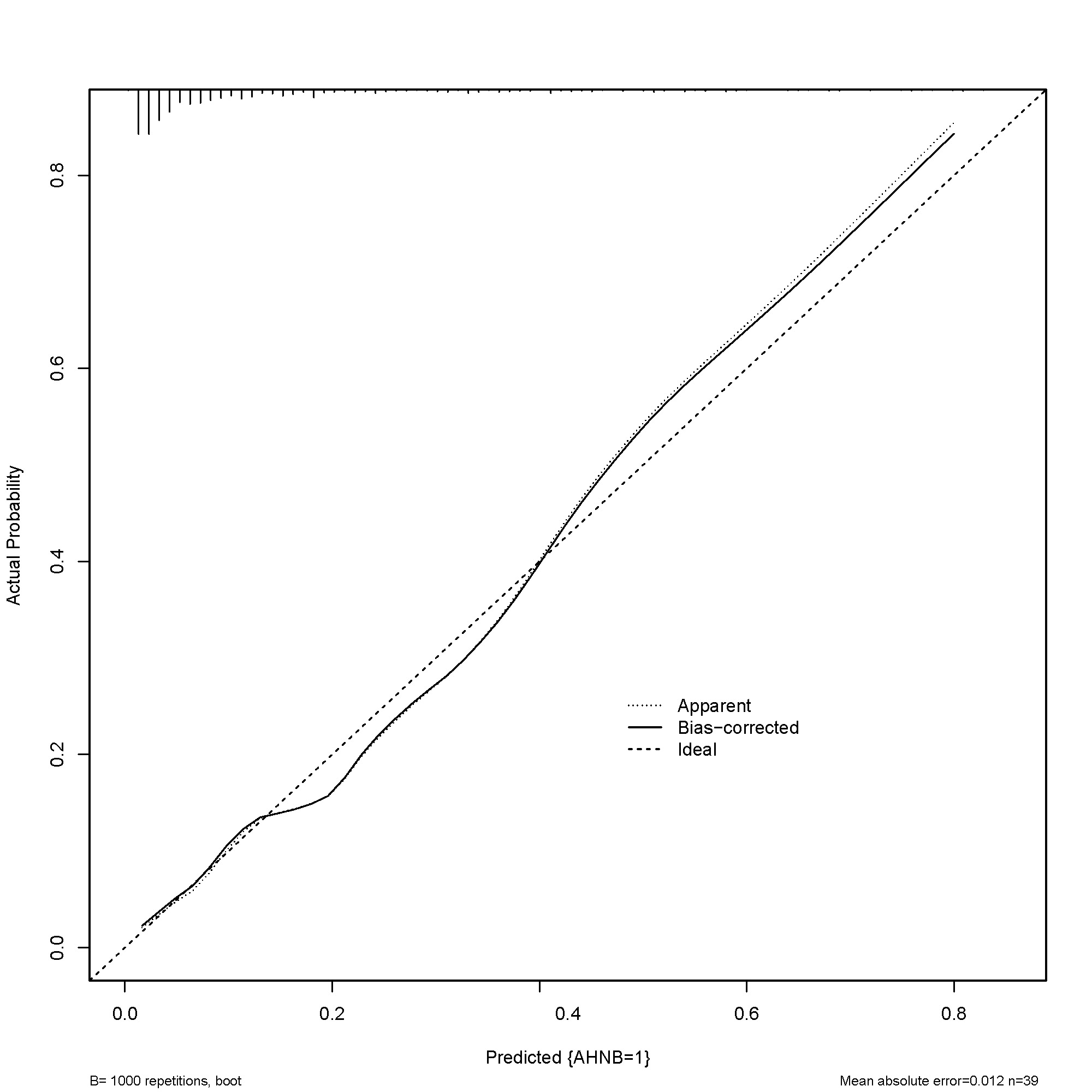


（a） （b）

Appendix Figure 9. Decision Curve Analysis of Nomogram for Predicting ALNB in Breast Cancer

(a: Training Set; b: Validation Set)


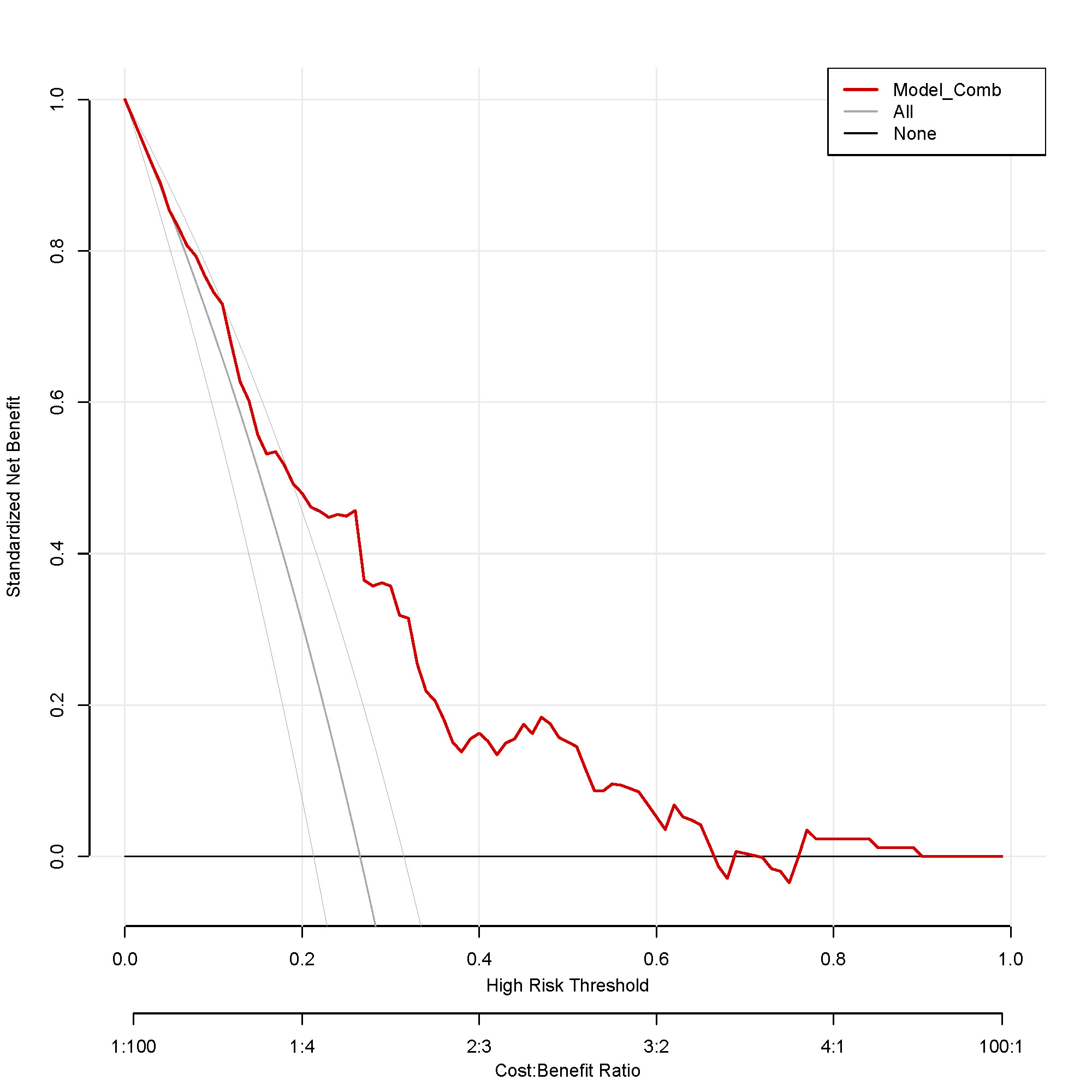

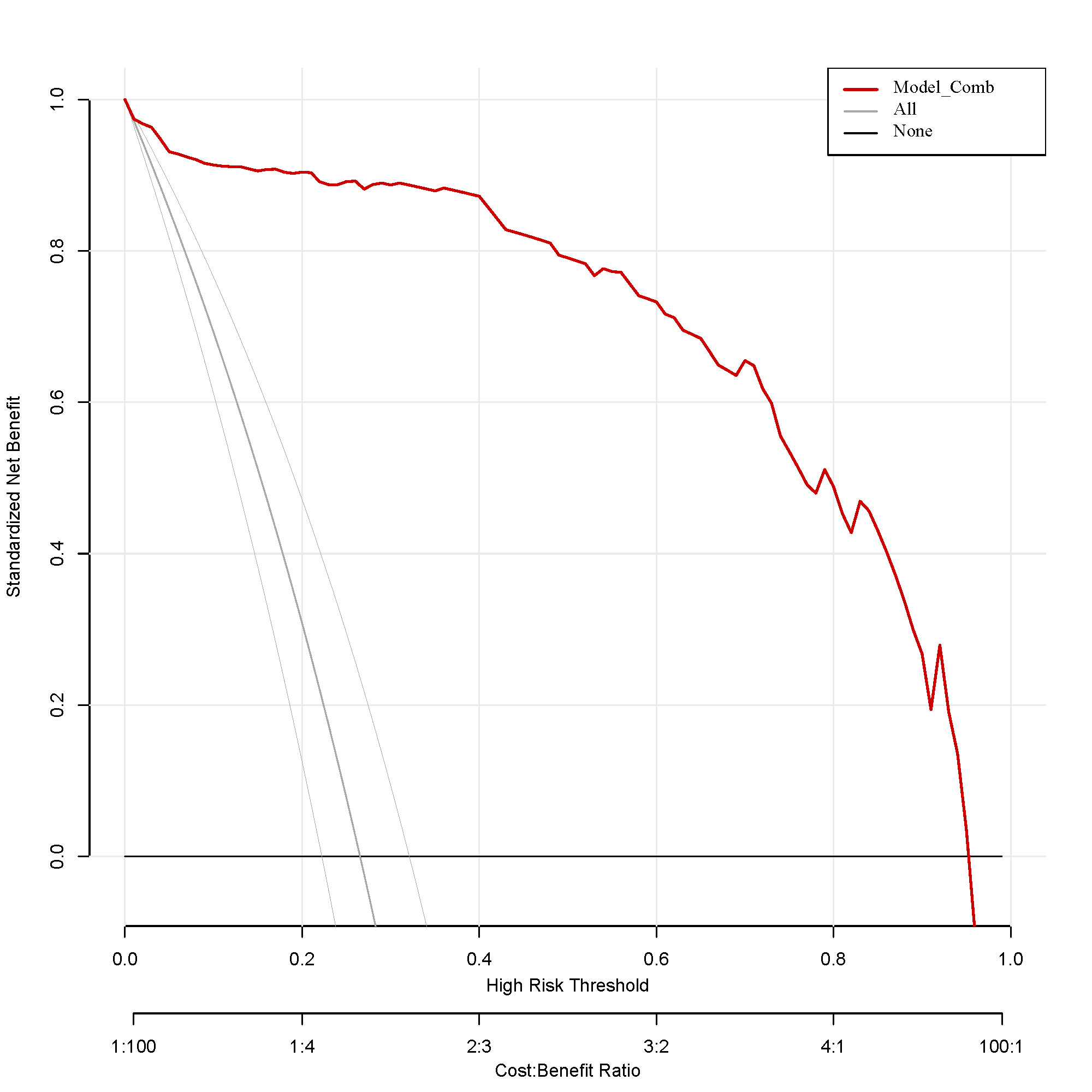


（a） （b）

Appendix Figure 10. Nomogram for Predicting ALNB in Breast Cancer


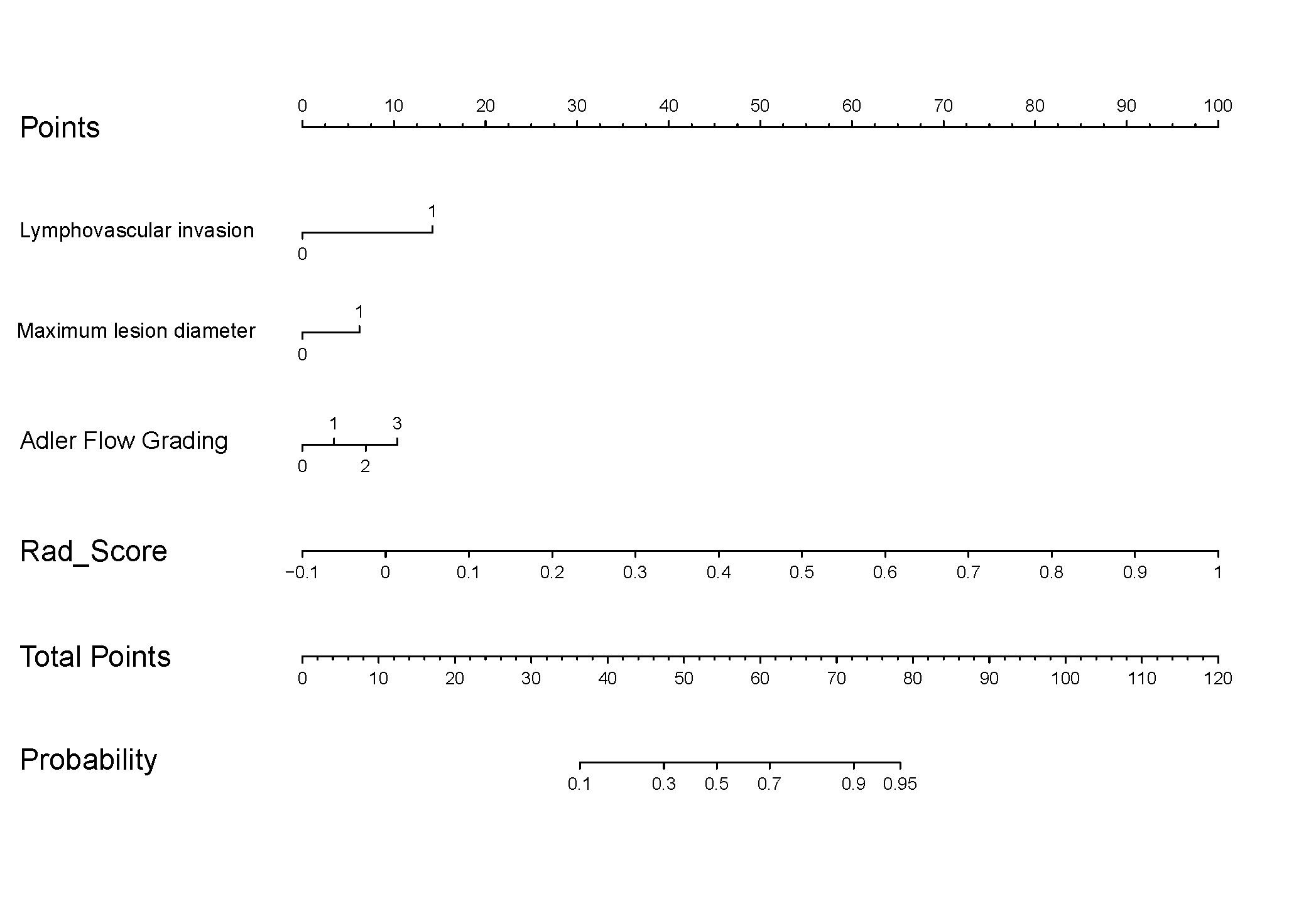

Supplement: Supplementary file 1 [file DataSheet1.docx]
